# Chemical Adsorption and Physical Confinement of Polysulfides with the Janus-faced Interlayer for High-performance Lithium-Sulfur Batteries

**DOI:** 10.1038/s41598-017-18108-0

**Published:** 2017-12-18

**Authors:** Poramane Chiochan, Siriroong Kaewruang, Nutthaphon Phattharasupakun, Juthaporn Wutthiprom, Thana Maihom, Jumras Limtrakul, Sanjog S. Nagarkar, Satoshi Horike, Montree Sawangphruk

**Affiliations:** 1 Department of Chemical and Biomolecular Engineering, School of Energy Science and Engineering, Vidyasirimedhi Institute of Science and Technology, Rayong, 21210 Thailand; 2 Department of Materials Engineering, School of Molecular Science and Engineering, Vidyasirimedhi Institute of Science and Technology, Rayong, 21210 Thailand; 3 Institute for Integrated Cell-Material Sciences (WPI-iCeMS), Institute for Advanced Study, Kyoto University, Yoshida, Sakyo-ku, Kyoto 606-8501 Japan

## Abstract

We design the Janus-like interlayer with two different functional faces for suppressing the shuttle of soluble lithium polysulfides (LPSs) in lithium-sulfur batteries (LSBs). At the front face, the conductive functionalized carbon fiber paper (*f*-CFP) having oxygen-containing groups i.e., -OH and -COOH on its surface was placed face to face with the sulfur cathode serving as the first barrier accommodating the volume expansion during cycling process and the oxygen-containing groups can also adsorb the soluble LPSs via lithium bonds. At the back face, a crystalline coordination network of [Zn(H_2_PO_4_)_2_(TzH)_2_]_n_ (ZnPTz) was coated on the back side of *f*-CFP serving as the second barrier retarding the left LPSs passing through the front face via both physical confinement and chemical adsorption (i.e. Li bonding). The LSB using the Janus-like interlayer exhibits a high reversible discharge capacity of 1,416 mAh g^−1^ at 0.1C with a low capacity fading of 0.05% per cycle, 92% capacity retention after 200 cycles and ca. 100% coulombic efficiency. The fully charged LSB cell can practically supply electricity to a spinning motor with a nominal voltage of 3.0 V for 28 min demonstrating many potential applications.

## Introduction

Lithium-sulfur batteries (LSBs) are of interest due to their high specific capacity (1675 mAhg^−1^) and specific energy (2600 WhKg^−1^). However, they inherently suffer from several drawbacks hindering their commercialization^1^. Firstly, the large volume change of the cathode during the cycling process can lead to short cycle life. This is because sulfur (S_8_) has a density of 2.03 g cm^−3^ but its fully discharged product, lithium sulfide (Li_2_S) has a density of 1.66 g cm^−3^. As a result, the volumetric expansion will increase up to 80% after fully discharged^2^. Secondly, the poor electrical conductivity of S_8_ (5 × 10^−30^ S cm^−1^) and Li_2_S (10^−13^ S cm^−1^) can lead to a low utilization of sulfur. When the insulating film of Li_2_S covers the entire surface of S_8_ electrode, the discharging reaction will be terminated due to its insulating property^3^. Also, the capacity of LSBs is dramatically decreased due to the pulverization of active material^4^ for which highly soluble intermediate substances namely lithium polysulfides (LPSs) can cause the shuttle effect. The LPSs formed on the cathode during cycling can freely diffuse between the cathode and anode thanks to a concentration gradient. Consequently, the active material and lithium metal anode are continuously consumed leading to poor discharge capacity and considerable capacity fading over long cycles^4^.

One of the best solutions used to address the drawbacks of LSBs is by inserting the interlayer or pseudo-upper current collector between the cathode and the separator or the functionalized the polymer separator, which can suppress the shuttle of LPSs and reduce the charge transfer resistance of the sulfur cathode^5–9^. For instance, Bai, S. *et al*. inserted the MOF@GO separator in LSBs which can selectively sieve lithium ions and effectively block the LPSs via MOF nano-pore^10^. Whilst, the GO can improve the brittleness of the MOF due to its mechanically flexible and robust structure. Peng, H.-J. *et al*. designed the nickel-iron layered double hydroxides (LDH) decorated on nitrogen doped graphene (NG) layer^11^. The lithiophilic NG and sulfiphilic LDH can improve the electrochemical performance by limiting the LPSs via the bifunctional effect. Peng, H.-J. *et al*. also demonstrated the use of Janus polymer separator for which the graphene framework was coated on the polypropylene membrane. The polypropylene acts as a conventional insulating separator preventing the contact between anode and cathode while the graphene framework can reactivate the shuttling LPSs, preventing the formation of inactive and insoluble species^12^. However, it is questionable about its practical use since it can increase the total weight of the current collector leading to overall low loading content of sulfur per cell or at least per cathode and interlayer weight. Another issue is that the interlayer or modified separator cannot wholly retard the shuttle of LPSs. Thus, the ideal interlayer needs to have high electrical conductivity, light weight, suitable pores for fast Li^+^ transportation, and adsorptive capacity of LPSs.

**Figure 1:**
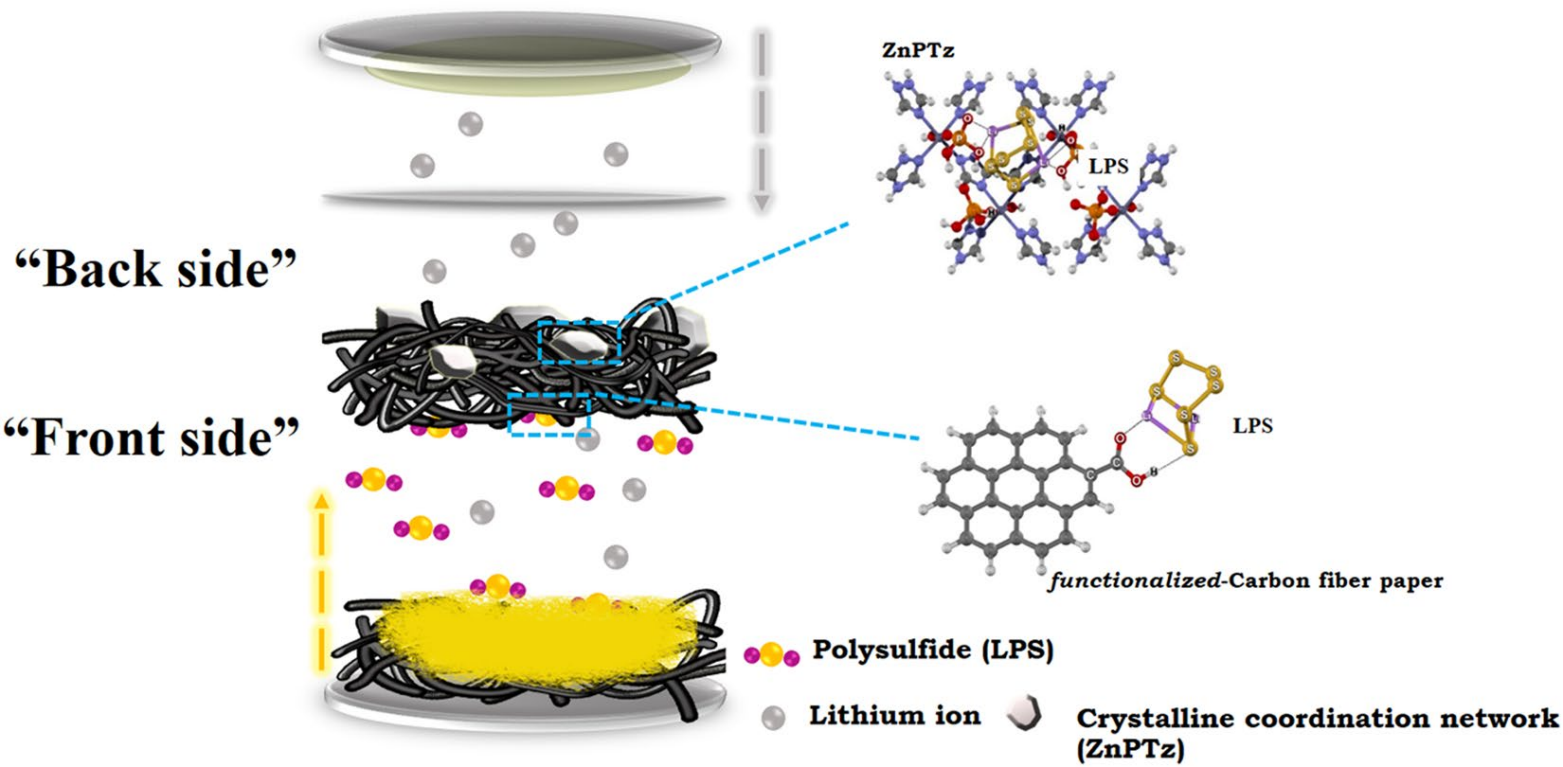
Schematic configuration of a LSB cell using the Janus interlayer with two functional surfaces (ZnPTz/*f*-CFP).

Herein, we introduce a new design of a single interlayer with “two functional faces” or “the Janus-like property” in LSBs having rather high loading sulfur content at the cathode. To the best of our knowledge, the Janus interlayer has not yet been used in LSBs. It is however necessary to note here that surface-modified separators for high-performance LSBs have been widely studied^13–15^. In this work, the first face of the Janus interlayer or the front side of the carbon fiber paper (CFP) interlayer is functionalized with oxygen-containing groups (i.e. -OH and -COOH) namely *f*-CFP. Note, the CFP is very light (ca. 2.5 mg/cm^2^), flexible, porous, and electrical conductive. The adsorption capacity of LPSs with the -OH and -COOH on *f*-CFP via Li bond^16^ were extensively investigated by density functional theory (DFT). Another face of the Janus interlayer or the back side of the *f*-CFP consists of an as-spray-coated two-dimensional (2D) coordination framework of octahedral Zn^2+^, 1,2,4-triazole (Tz), and orthophosphates with a formula of [Zn(H_2_PO_4_)_2_(TzH)_2_]_n_ namely ZnPTz^17^. The rich oxygen atoms with lone-pair electrons of phosphate groups on the surface of ZnPTz can form a strong lithium bond interaction for binding LPSs. In addition, the dense and non-porous structure of ZnPTz can serve as a physical shield for blocking the shuttling effect of LPSs^18^. Therefore, the ZnPTz was chosen as the interlayer material because it can suppress LPSs by both physical and chemical interaction. The front side of *f*-CFP is placed face to face with the cathode. Whilst, the back side with ZnPTz is placed face to face with the polymer separator as illustrated in Fig. 1. For comparison, the opposite configuration was also fabricated and investigated.

This design aims to use the front side of *f*-CFP with high electrical conductivity for reducing the charge transfer resistance of the sulfur cathode and the -OH and -COOH groups as the first barrier for mitigating volume expansion, trapping LPSs, and improving electron transfer^19^. The oxygen-containing groups on the surface of *f*-CFP can trap the LPSs via a lithium bonding. The interconnected fiber network can accommodate the large volume expansion of sulfur during discharging. A further design on the back side of *f*-CFP with ZnPTz will serve as the second barrier adsorbing the left LPSs passing through the *f*-CFP layer. At the back side, the oxygen atoms of the mono-coordinated phosphate groups in the unique structure of ZnPTz can form a chemical interaction with two lithium atoms of LPSs. Also, ZnPTz particles can physically reduce the porosity between adjacent fibers of CFP suppressing the permeability of polysulfides via a physical confinement. With our design, the as-fabricated LSB exhibits an excellent initial discharge capacity of 1,416 mAh g^−1^ at 0.1C after the removal of the capacity contributed from non-related active materials. The LSB has 92% capacity retention after 200 cycles and ca. 100% coulombic efficiency.

## Results and Discussion

### Morphologies of the as-synthesised materials

**Figure 2:**
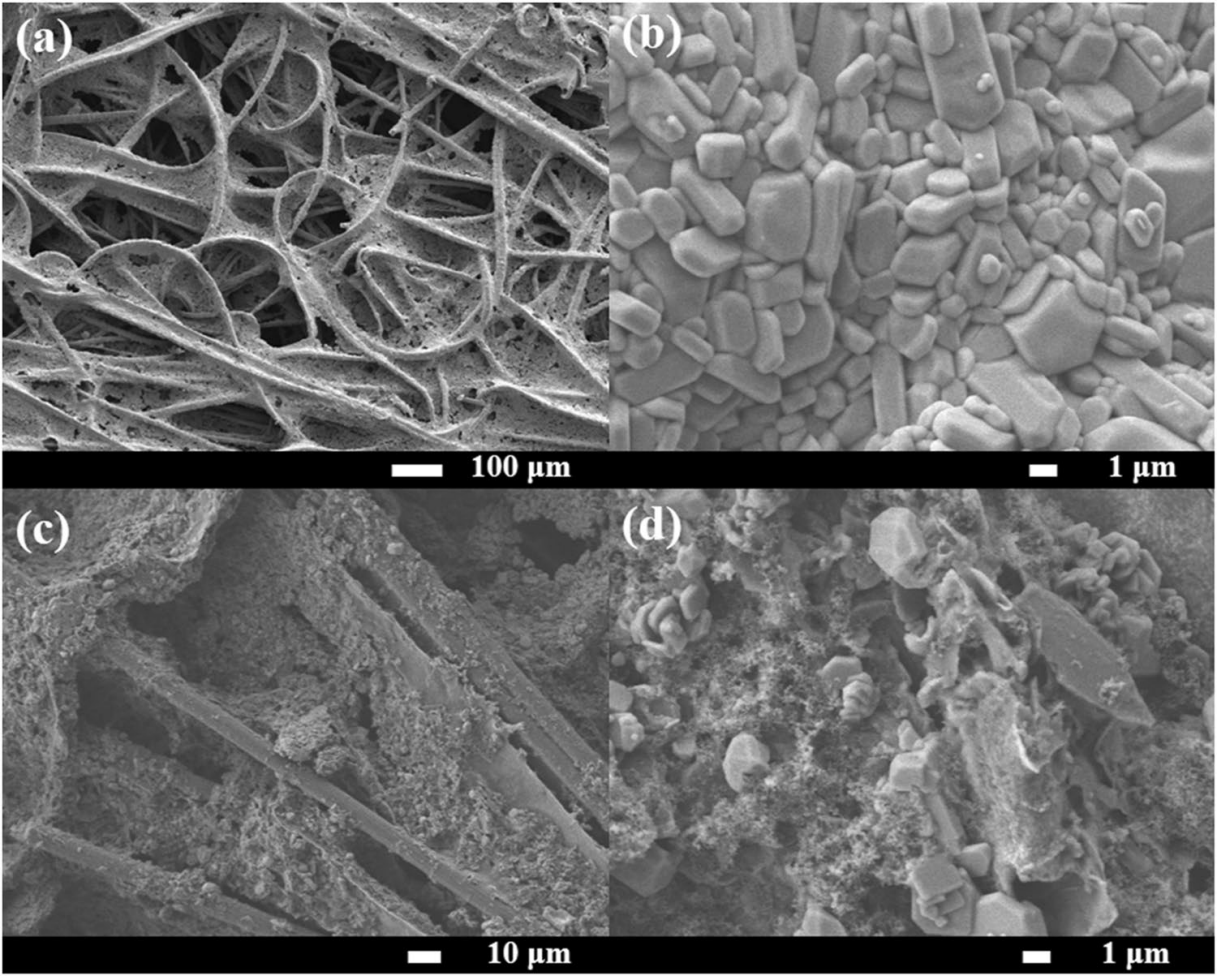
FESEM images of (**a**) *f*-CFP, (**b**) ZnPTz powder as well as (**c**) low- and (**d**) high-magnification FESEM image of the back side of the ZnPTz/*f*-CFP interlayer.

An FESEM image in Figure S1 shows that the pristine CFP (ca. 2.5 mg cm^−2^) has a fiber structure with a diameter of ca. 8 μm forming an interconnected conductive network with high porosity and flexibility^20,21^. The fibers were linked together with polytetrafluoroethylene (PTFE) adhesive, which is typically used in the manufacturing process of CFP. The internal voids generating from the connected fiber are expected to accommodate the volume expansion during the discharging process which can prevent the pulverization of active material^22^. After acid treatment in a mixed acid of conc. H_2_SO_4_ and HNO_3_ in a volume ratio of 3:1 at 60 °C for 1 h, oxygen-containing groups i.e. -OH and -COOH were functionalized to the surface of CFP so-called *f*-CFP (ca. 2.4 mg cm^−2^)^23^. The FESEM image (Fig. 2a) shows no significant change compared to the pristine CFP. This implies that the functionalization process does not destroy its fiber structure/network, but providing a uniform distribution of oxygen functionalities as seen in an EDX mapping in Figure S2. The FESEM image of ZnPTz powder in Fig. 2b indicates its nonporous feature with an irregular shape with particle size ranging from 1–4 μm. The morphology of ZnPTz coated on the back side of *f*-CFP interlayer is also shown in Fig. 2c and d. It exhibits a good dispersion of ZnPTz for which most interspaces of *f*-CFP were occupied by ZnPTz particles. EDX analysis was also carried out as shown in Figure S3 of the supporting information.

### Morphologies of the cycled interlayers

**Figure 3:**
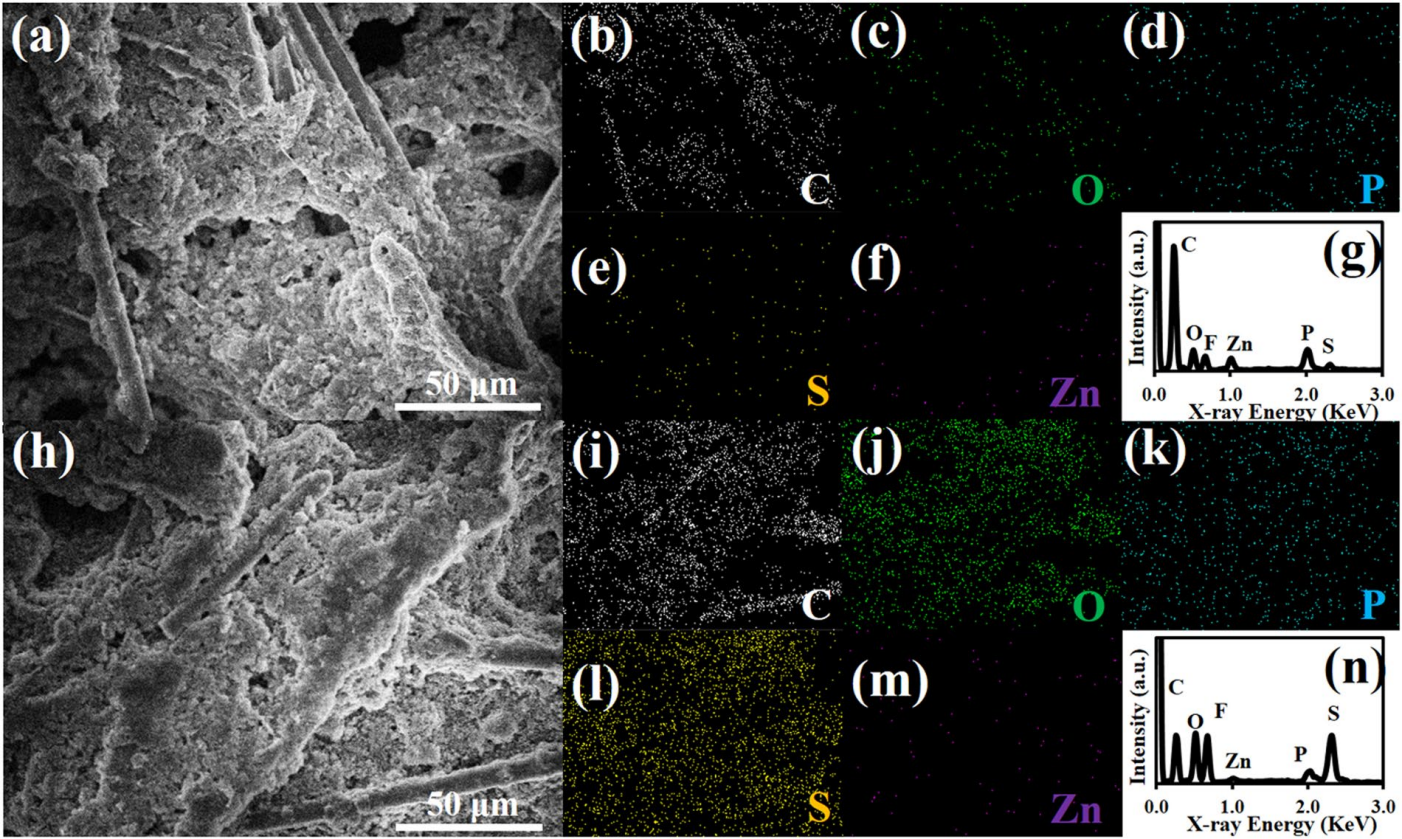
(**a**) FESEM image, EDX mapping of (**b**) carbon, (**c**) oxygen, (**d**) phosphorus (**e**) sulfur, (**f**) zinc, and (**g**) EDX spectrum of as-tested ZnPTz/f-CFP interlayer or the back side of the interlayer and (**h**) FESEM image, EDX mapping, (**i**) carbon, (**j**) oxygen, (**k**) phosphorus (**l**) sulfur, (**m**) zinc, and (**n**) EDX spectrum of the front side of the interlayer.

To further confirm the stability of ZnPTz/*f*-CFP interlayer, the cell after the stability test for 200 cycles was disassembled and characterized by FESEM and EDX. Figure 3a shows an FESEM image of the as-tested ZnPTz on the back side of the interlayer. There is no significant change on the morphology when compared with that of ZnPTz/*f*-CFP interlayer before tested (Figure S3a). In addition, the EDX mapping and spectrum (Fig. 3b–g) also reveal element on its surface for which small amount of sulfur is found confirming that the interlayer can effectively suppress LPSs. At the front side of the interlayer, an FESEM image (Fig. 3h) and EDX mapping and spectrum (Fig. 3i–n) show that the carbon fibers were mostly covered with sulfur species due the strong adsorption of LPSs at the oxygen-containing groups of f-CFP. For comparison, both sides of the *f*-CFP interlayer were also investigated by FESEM and EDX as shown in Figures S4 and S5. As a result, sulfur is clearly found on both sides of the f-CFP interlayer due to its too high internal porosity in micrometer scale located between adjacent carbon fibers. Thus, the *f*-CFP itself cannot entirely trap all LPSs.

### Structures of the as-synthesised materials

To examine the structure of the as-prepared samples, FTIR and RAMAN were investigated as shown in Figures S6 and S7, respectively. An FTIR spectrum of *f*-CFP in Figure S4 indicates three main peaks at 3500, 1200, and 1700 cm^−1^ corresponding to O-H, C-O, and C=O bonds, respectively. For the FTIR spectra of ZnPTz powder and ZnPTz/*f*-CFP interlayer (Figure S7a), the peaks at 2500–3500 cm^−1^ can be assigned to N-H bond of triazole in the ZnPTz frameworks. Whilst, the peaks of Zn-O, Zn-N, and phosphoric groups can be seen around 500–1600 cm^−1^.^24^ For the Raman result (Figure S7b), the feature of Zn-O can be seen at 300–600 cm^−1^. Whilst, the triazole and phosphate groups can be observed at the peaks around 900–1500 and 3200 cm^−1^^25^. Both FTIR and Raman confirm that the crystalline coordination networks and the functionalities of ZnPTz remain unchanged after it was coated on the *f*-CFP. In addition, the XRD patterns of the materials are also shown in Figure S8^17^.

### Structures of the cycled interlayers and separators

**Figure 4:**
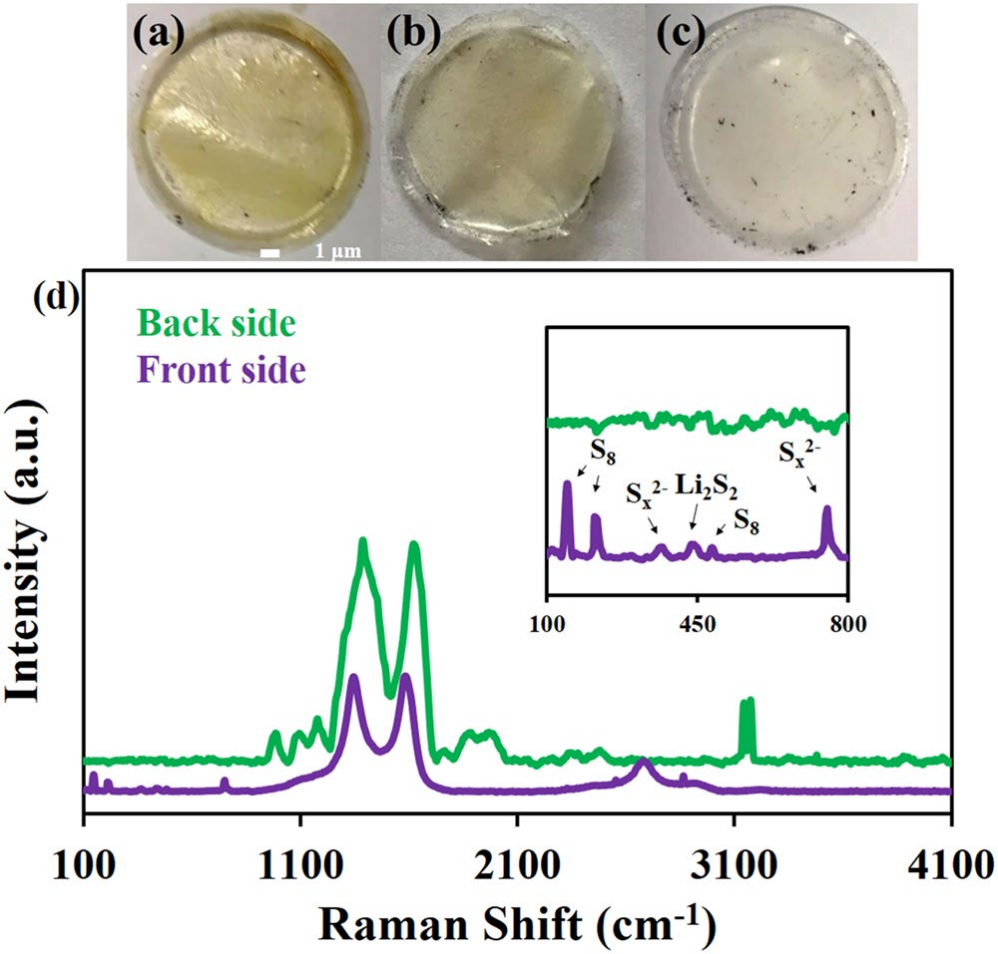
The as-tested separator of LSB over 200 cycles; (**a**) without interlayer and (**b**) with f-CFP interlayer, (**c**) with ZnPTz/f-CFP interlayer as well as (**d**) the RAMAN spectra of the back side (green line) and the front side (purple line) of the interlayer.

In addition, the polymer separator of each cell after the cycling test was investigated by optical microscopy as shown in Fig. 4a–c. Figure 4a shows the photograph of the separator from the cell without using the interlayer. The LPSs can freely diffuse between anode and cathode leading to the dark yellow color of LPSs on the photograph. When the *f*-CFP interlayer was employed, the LPSs can be adsorbed by the interlayer. In another word, the *f*-CFP can partially block the soluble substances by means of physical and chemical interaction. However, the light yellow of LPSs can still be observed as shown in Fig. 4b implying that some LPSs can still penetrate through the interlayer. For the ZnPTz/*f*-CFP interlayer, the as-tested separator displays almost white in color (Fig. 4c) without LPSs. Furthermore, both sides of the as-tested ZnPTz/*f*-CFP interlayer were investigated by RAMAN (Fig. 4d). A RAMAN spectrum of the back side of the ZnPTz/*f*-CFP interlayer demonstrates the characteristic of ZnPTz without any peaks of sulfur species (green line). Whilst, a RAMAN spectrum of the front side of the interlayer (purple line) displays major peaks at 1,632 and 1,613 cm^−1^ which are the characteristics of the *f*-CFP as well as the signals of octasulfur, LPSs, and lithium disulfide^26–28^. These results suggest that the ZnPTz can effectively reduce the shuttle effect, which is one of the major problems of the LSBs. Besides, the XRD spectrum of the as-cycled interlayer (Figure S8) remains unchanged confirming that the crystalline coordination network is stable over 200 cycles.

### Electrochemical evaluation of the as-fabricated batteries

For the electrochemical evaluation, most previous publications normally reported the rate performance of LSBs based on sulfur active materials without subtracting the capacity contribution from the current collector, host material, and interlayer resulting in the overestimated results. Therefore, the effects of non-active materials were deducted in this work by fabricating the LSB cells without the sulfur active material. These cells were tested in the same conditions as those of the LSBs in which the rate performance from non-active material cells was used to subtract the result from each typical LSB cell as shown in Figures S9 and S10. All the results exhibit high capacity at the first discharge and the following cycles dramatically decrease. This can be ascribed by the decomposition of electrolytes, forming the solid electrolyte interphase (SEI) on the surface of lithium metal anode^2,29^.

**Figure 5:**
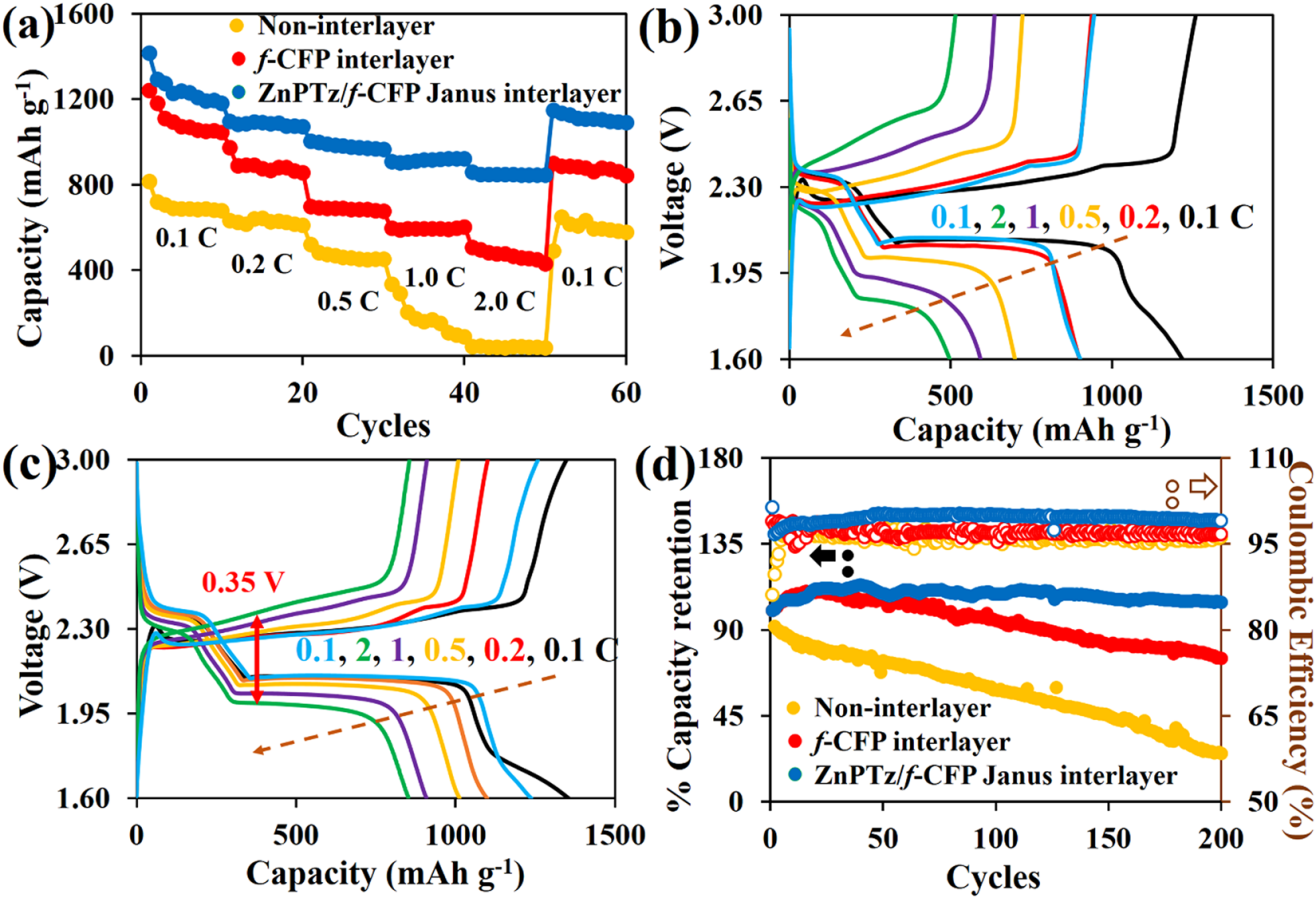
(**a**) The rate performance of LSBs with and without interlayer and the charge-discharge voltage profiles of LSBs with (**b**) *f*-CFP interlayer and (**c**) ZnPTz/*f*-CFP interlayer, and (**d**) the cycling performance of LSBs with different interlayers. All specific capacities were calculated based on the mass of sulfur after deducting non-active material contribution.

Figure 5a shows the specific capacities of LSBs at various applied current densities from 0.1–2.0C. The LSB with the ZnPTz/*f*-CFP interlayer delivers the highest reversible specific capacity of 1,416 (84.5% sulfur utilization), 1,098, 1,004, 910, and 859 mAh g^−1^ at 0.1, 0.2, 0.5, 1.0, and 2.0 C, respectively. When the current was turned back to 0.1C after high rate testing, the use of the ZnPTz/*f*-CFP interlayer can maintain its specific capacity, indicating excellent reversibility and stability of the cell. Note, the effect of ZnPTz mass loading contents (1.5, 2.0, 2.5, and 3.0 mg cm^−2^) was also investigated as shown in Figure S11 for which the optimum loading content of ZnPTz is 2.5 mg cm^−2^. In addition, the cell with high sulfur loading content (9.57 mg cm^−2^ or 19.14 mg a cell) was also fabricated and investigated using the ZnPTz/*f*-CFP interlayer (Figures S14 and S15) demonstrating the practical use of the LSB. The cell exhibits the initial discharge capacity of 1,082, 909, 760, 215, 70, and 757 mAh g^−1^ at 0.1, 0.2, 0.5, 1.0, 2.0, and 0.1 C, respectively.

The GCD profiles of LSBs using *f*-CFP and ZnPTz/*f*-CFP interlayers at each current rate are shown in Fig. 5b and c, respectively. The voltage profile of LSB typically exhibits two voltage plateaus associated with the formation of high-order LPSs (Li_2_S_X_, 4 < X < 8) at 2.3 V and low-order LPSs (Li_2_S_2_ and Li_2_S) at 2.1 V^4,30,31^. All the charge-discharge voltage profiles clearly exhibit two plateau regions implying an electrochemically reversible reaction^32,33^. However, the LSB using *f*-CFP interlayer exhibits higher polarization (ΔE) than that of the cell using ZnPTz/*f*-CFP interlayer when the current rates are increased. The voltage gap between the reduction and oxidation of ZnPTz/*f*-CFP interlayer is 0.16 V at 0.1C and sustains at 0.35 V at the highest current rate of 2.0C thanks to its fast kinetics. This is due to the small amount of long-chain LPSs on its surface^32^. In addition, the polarization of the LSB using the ZnPTz/*f*-CFP interlayer is lower than that of other previous report^32^. This is a major advantage of using the ZnPTz/*f*-CFP interlayer. The stability test of LSBs was also investigated using GCD at 0.5C from 1.6–3.0 V for 200 cycles as shown in Fig. 5d. Not only can the ZnPTz/*f*-CFP interlayer provide a good rate capability but also it shows a promising cycling performance. Note that the capacity of LSBs using the interlayer normally increases during the first period (ca. 1–40 cycles) because the LPSs dissolved into the electrolyte can deposit at the vacancy site of the interlayer, leading to the increasing amount of sulfur species in the discharge reaction^34^. After 200 cycles, the cell with the ZnPTz/*f*-CFP interlayer shows a capacity retention of 92% as compared with the highest discharge capacity at the 40^th^ cycle with a fading rate of 0.05% and a coulombic efficiency of 98%. For comparison, the LSB cell using the *f*-CFP interlayer exhibits a capacity retention of 71.8% as compared with the highest discharge capacity at the 23^rd^ cycle with a fading rate of 0.16%. Note, the cell without using interlayer displays a dramatic decrease in capacity. Furthermore, in the case of high sulfur content of 9.57 mg cm^−2^ (19.14 mg S a cell) with ZnPTz/*f*-CFP interlayer (Figure S15), the capacity retention remains 89% with a coulombic efficiency over 96% after tested at 0.1 C for 50 cycles. Note, this loading is much higher than other previous report^35^. In addition, the practical application of the LSB cell with the ZnPTz/*f*-CFP interlayer was demonstrated for which the fully charged LSB can supply the electricity to a spinning motor with a nominal voltage of 3 V over 28 min (see the video in the supporting information).

**Figure 6:**
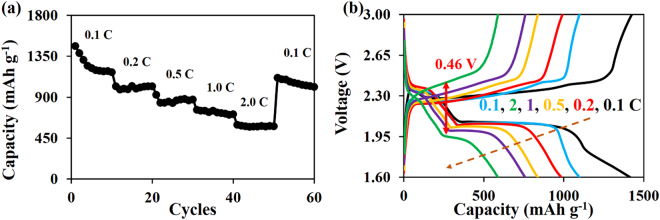
(**a**) Rate performance and (**b**) charge-discharge voltage profile of the LSB using the opposite configuration of the Janus interlayer at various current densities.

For comparison, the LSB using the opposite configuration of the Janus interlayer was also fabricated for which the front side of the interlayer is the ZnPTz-coated *f*-CFP and the back side is the *f*-CFP. The result shows that the rate performance of the LSB is much lower than the LSB using the Janus interlayer with the front side of *f*-CFP and the back side of the ZnPTz-coated *f*-CFP (see Fig. 6). This is because the surface of ZnPTz-coated *f*-CFP has lower electrical conductivity when compared to that of *f*-CFP. Thus, the ZnPTz-coated *f*-CFP surface cannot wholly electrically contact with the cathode leading to poor sulfur utilization^36,37^. This can be supported by charge-discharge voltage profile as shown in Fig. 6b. The polarization is 0.22 V at 0.1C, greater than 0.19 V at 0.1C of the *f*-CFP interlayer. On the other hand, the polarization was maintained at 0.46 V at 2.0C, which is lower than that of the *f*-CFP due to the strong chemical interaction between the ZnPTz and LPSs. Therefore, the first barrier or front side of the interlayer should be the high conductive side, *f*-CFP and the back side should be the high chemical adsorptive material, ZnPTz. This strategy would gain the highest benefit of the as-designed Janus interlayer.

**Figure 7:**
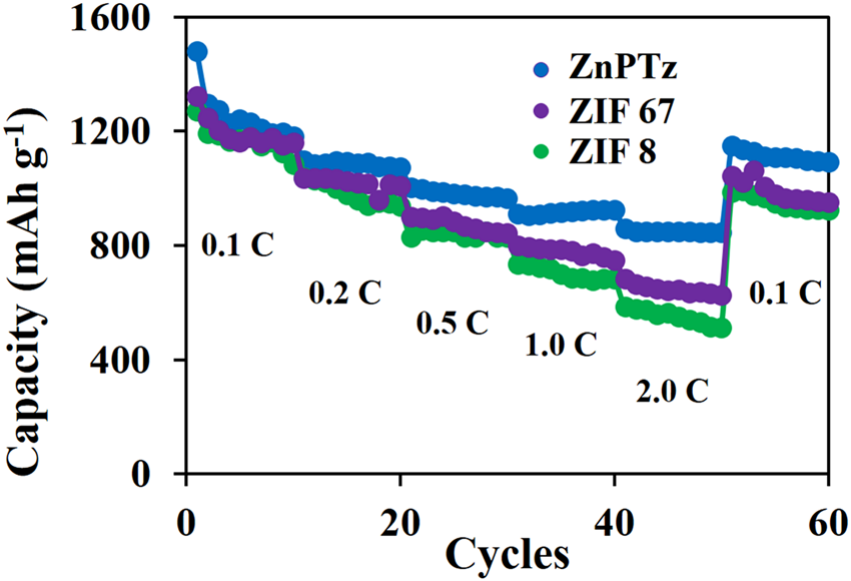
The rate performance of LSBs using ZnPTz/*f*-CFP, ZIF 67/*f*-CFP, and ZIF 8/*f*-CFP interlayers.

To further clarify the intrinsic properties of ZnPTz for binding the LPSs, other related interlayers including ZIF-8 (zinc ions coordinated with four imidazolate rings) and ZIF-67 (cobalt ions coordinated with four imidazolate rings) were also investigated and compared for which the cluster atoms can bind LPSs by a Lewis acid-base interaction^38,39^. Note that the surface morphologies of the ZIF-8 and ZIF-67 coated on *f*-CFP are shown in Figure S13. The LSB with the ZIF-67/*f*-CFP interlayer (Fig. 7) provides the discharge capacities of 1,322, 1035, 899, 798, 683, and 1,043 mAh g^−1^ at 0.1, 0.2, 0.5, 1.0, 2.0, and 0.1C, respectively. Whilst, the use of ZIF-8/*f*-CFP exhibits the discharge capacities of 1,270, 1037, 828, 736, 585, and 985 mAh g^−1^ at 0.1, 0.2, 0.5, 1.0, 2.0, and 0.1C, respectively. ZIF-8 exhibits lower rate capability when compared with ZIF-67 due to its less Lewis acid-base interaction. Whilst, the chemical interaction from ZnPTz can provide the highest electrochemical performance.

### DFT calculation of the binding energy between LPSs and interlayers via lithium bonds

**Figure 8:**
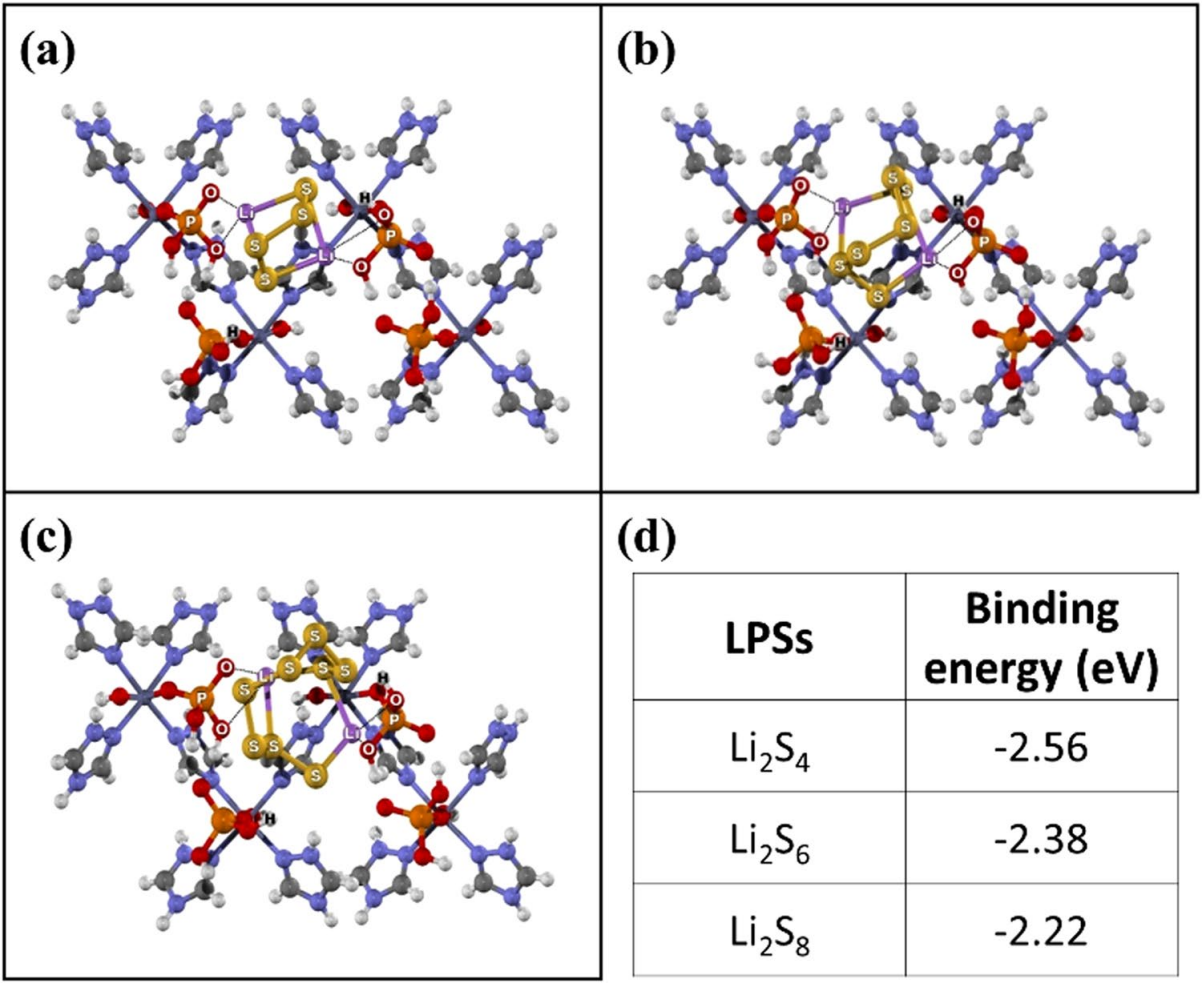
Optimized structures of (**a**) Li_2_S_4_, (**b**) Li_2_S_6_, and (**c**) Li_2_S_8_ adsorbed over ZnPTz and (**d**) the binding energy of LPSs and ZnPTz.

To further understand the interaction between LPSs and interlayers, the DFT calculation was used to evaluate the adsorption of soluble LPS substances (Li_2_S_4_, Li_2_S_6_, and Li_2_S_8_) on the functional groups of the interlayers. The *f*-CFP consists of the oxygen-containing functional groups (i.e., -OH and -COOH) on its surface which can interact with the LPSs. In the case of the hydroxyl groups, the LPSs can be adsorbed by the lone pair electron of oxygen via Li bonds^16^ (Figure S16) with the binding energies of −1.07, −1.03, and −1.10 eV which can be ascribed to Li_2_S_4_, Li_2_S_6_, and Li_2_S_8_, respectively. Whilst, the O and H from the carboxylic groups can interact with Li and S forming the bi-dentate structure (Figure S17) with the binding energies of −0.71, −0.78, and −0.91 eV from Li_2_S_4_, Li_2_S_6_, and Li_2_S_8_, respectively. For the ZnPTz 2D coordination network interlayer, the binding of LPSs on the surface of ZnPTz owing to the interaction between two lithium atoms of LPSs and the oxygen atoms of the phosphate groups play an important role to enhance the capacity retention of LSB. Also, the strong interaction can be formed between LPSs and phosphate groups (Fig. 8a–c). The NBO analysis reveals the electron transfer from the adsorbent to the LPSs that the partial charge of the adsorbed LPSs slightly becomes negative. As a result, the calculated binding energies of Li_2_S_4_, Li_2_S_6_, and Li_2_S_8_ are −2.56, −2.38, and −2.22 eV, respectively as shown in Fig. 8d. Note that the interaction energies of these high-order LPSs are stronger than those reported for LPSs bound to other materials such as doped carbons, graphitic carbon nitride, and functionalized polymers^40^. For the other metal-coordination frameworks (ZIF-67 and ZIF-8), they can limit LPSs by the Lewis acid-base interaction between the metal active sites and LPSs (Figures S20 and S21). The ZIF-67 shows higher electronegativity (EN) than ZIF-8 resulting in greater binding energy than ZIF-8. Note that the calculated binding energies of LPSs with the functional groups at the interlayers are listed in Table S1. These results are in good agreement with the rate capability result as shown in Fig. 7. The ZnPTz interlayer exhibits the best rate performance. Whilst, the ZIF-8 interlayer shows the lowest rate capability.

## Conclusions

In summary, we design a new Janus-like interlayer with two different functional faces or sides for suppressing the shuttle of polysulfides in lithium-sulfur batteries. At the front side, the conductive functionalized carbon fiber paper (*f*-CFP) having oxygen-containing groups i.e., -OH and -COOH on its surface was placed face to face with the sulfur cathode serving as the first barrier accommodating the volume expansion during cycling process and the oxygen-containing groups can also adsorb the soluble lithium polysulfides (LPSs) via lithium bonds. At the back side, a crystalline coordination network of [Zn(H_2_PO_4_)_2_(TzH)_2_]_n_ (ZnPTz) was coated on the back side of *f*-CFP serving as the second barrier retarding the left LPSs, which can pass through the f-CFP layer via both physical confinement and chemical adsorption. The insulating ZnPTz particles on the back side can physically reduce the porosity of CFP by occupying the air voids between adjacent fibers suppressing the permeability of polysulfides via a physical confinement. A strong interaction via Li bonding between two lithium atoms of LPSs and oxygen atoms of the phosphate groups of ZnPTz also plays a major role for anchoring the left LPSs. The LSB using the Janus-like interlayer exhibits a high reversible discharge capacity of 1,416 mAh g^−1^ at 0.1C with a low capacity fading of 0.05% per cycle, 92% capacity retention after 200 cycles and ca. 100% coulombic efficiency. The fully charged LSB cell can practically supply electricity to a spinning motor with a nominal voltage of 3.0 V for 28 min demonstrating many potential applications.

## Experimental Section

### Synthesis of ZnPTz

ZnPTz was synthesized by following our previous report^17^. Briefly, 81 mg zinc oxide, 138 mg 1,2,4-triazole (Tz), and 134 μl phosphoric acid were transferred to a 10-ml Teflon jar with two steel-cored 10-mm Teflon balls and then ground for 6 min in a Retch MM200 grinder mill operated at 25 Hz. The mixture was washed with methanol and evacuated at 100 °C overnight to obtain the powder in a dry pure phase. The characterization was conducted by thermogravimetric analysis (TGA) and powder X-ray diffraction (XRD)^17^.

### Preparation of an interlayer with two functional surfaces (Janus interlayer)

Carbon fiber paper (CFP) with a thickness of 400 μm and an electrical resistance of < 12 MΩ cm^−2^ (SGL CARBON SE, Germany) was firstly soaked in the mixed solution of H_2_SO_4_ and HNO_3_ in a volume ratio of 3:1 at 60 °C for 1 h. The CFP was washed with DI water several times and dried at 60 °C overnight to obtain the functionalized-CFP (*f*-CFP)^23,41^. To prepare the Janus interlayer (ZnPTz/*f*-CFP), ZnPTz was mixed with super P and polyvinylidene fluoride (PVDF) in a weight ratio of 8:1:1 in *N*-methyl-pyrrolidone to form a slurry. The homogeneous mixture was spray-coated on the back side of *f*-CFP with a diameter of 2.0 cm (Paasche Airbrush Company, USA)^20^. Note that the mass loading of ZnPTz on the *f*-CFP was varied from 1.0–3.0 mg cm^−2^.

### Characterizations

The morphology and element of the as-prepared samples were characterized by Field Emission Scanning Electron Microscopy (FESEM) and Energy-dispersive X-ray spectroscopy (EDX) using JEOL (JSM-7610F) instrument operated at 1 kV. The structure of all samples was examined by RAMAN using a Senterra Dispersive Raman Microscope (Bruker) with an excitation wavelength of 532 nm, Fourier transform infrared spectroscopy (FTIR) using PerkinElmer instrument, and powder X-ray diffraction (PXRD, Bruker, Germany) using a monochromatic Cu Kα radiation (λ = 0.15405 nm). The as-tested electrodes and interlayers disassembled were washed several times with a DOL/DME mixture in a volume ratio of 1:1 and dried in a vacuum oven at 60 °C overnight prior to the *ex situ* FESEM-EDX, RAMAN, and XRD measurement.

### Fabrication of LSBs and the electrochemical evaluation

The cathode was prepared by mixing sulfur, carbon black, and PVDF in a weight ratio of 8:1:1 in NMP forming a black slurry. The homogeneous slurry was coated on the CFP substrate with a diameter of 1.4 cm and dried at 60 °C overnight. The cathode electrode was then pressed by a rolling machine (Gelon) to adjust the electrode thickness prior to the fabrication. The mass of sulfur loading on the CFP is ca. 3.5 mg cm^−2^. The coin cells were assembled in an argon-filled glovebox (Mbraun, Germany) using CR2032 coin cell in which the lithium metal and Celgard 2400 film were used as an anode and a separator, respectively for which the ZnPTz/*f*-CFP interlayer was inserted between the cathode and the separator.1.0 M lithium bis(trifluoromethane) sulfonamide (LITFSI) with 0.1 M lithium nitrate (LiNO_3_) additive dissolved in a mixture of 1,3-dioxalane (DOL) and dimethoxymethane (DME) with a volume ratio of 1:1 was used as an electrolyte. The LSB performance was examined by Galvanostatic charge-discharge technique (GCD) at different current densities from 0.1 to 2 C (1C=1,675 mAh g^−1^) in a potential range of 1.6–3.0 V using a NEWARE battery tester.

### Computational methods

All calculations were performed using the M06–2X density functional^42^ with Q-CHEM 4.4 software^43^. The 6–31 G(d,p) basis set was employed for H, Li, C, N, O, S, and P atoms while Zn atom was described with LANL2DZ pseudopotential basis set^44^. The unit cell of the [Zn-(H_2_PO_4_)_2_(TzH)_2_]_n_ coordination network^17^ is illustrated in Figure S19 of the supporting information. It consists of octahedral Zn^2+^, orthophosphate and 1,2,4-triazole forming two-dimensional (2D) sheets in the ab plane. The sheet stacks each other in c direction via the hydrogen bond of phosphate groups in the layers (Figure S19a). To simulate the interaction of LPSs on the surface and reduce the computational cost, the simplest model representing the 001 surfaces was used as shown in Figure S19b. This model composes of four Zn atoms mono-coordinated with phosphate groups and 1,2,4-triazole. For comparison, ZIF-8 and ZIF-67 (Figure S21) containing one metal atom (Zn for ZIF-8 and Co for ZIF-67) coordinated with three 2-methylimidazolate ligands were also utilized in this work. The models of the materials used in the calculation were comparable with those in other previous work^45^. For the *f*-CFP, the model was constructed via the functionalization at the edge of the CFP. The model consists of 24C atoms and 12H atoms as well as carboxyl (-COOH) or hydroxyl (-OH) are at the edge of the carbon model as shown in Figure S18 ^46^. Partial charges and population analysis were determined by the natural bond orbital (NBO) method^47^. The calculated binding energy (*ΔE*) of lithium polysulfides (Li_2_S_x_; x = 4, 6, and 8) on the coordination network and *f*-CFP was defined as follows:

$${\rm{\Delta }}E=E(L{i}_{2}{S}_{x}-adsobates)-E(L{i}_{2}{S}_{x})-E(adsorbates)$$

where *E*(*Li*_2_*S*_*x*_*-adsobates*), *E*(*Li*_2_*S*_*x*_), and *E*(*adsorbates*) are the total energies of the adsorbed Li_2_S_x_ on ZnPTz, the isolated Li_2_S_x_, and the CFP model, respectively. Basis set superposition error (BSSE) correction was used to obtain more reliable interaction energies.

## Electronic supplementary material

Supporting information
