# Author Correction: Chemical Adsorption and Physical Confinement of Polysulfides with the Janus-faced Interlayer for High-performance Lithium-Sulfur Batteries

**DOI:** 10.1038/s41598-018-24801-5

**Published:** 2018-06-01

**Authors:** Poramane Chiochan, Siriroong Kaewruang, Nutthaphon Phattharasupakun, Juthaporn Wutthiprom, Thana Maihom, Jumras Limtrakul, Sanjog S. Nagarkar, Satoshi Horike, Montree Sawangphruk

**Affiliations:** 1 Department of Chemical and Biomolecular Engineering, School of Energy Science and Engineering, Vidyasirimedhi Institute of Science and Technology, Rayong, 21210 Thailand; 2 Department of Materials Engineering, School of Molecular Science and Engineering, Vidyasirimedhi Institute of Science and Technology, Rayong, 21210 Thailand; 3 Institute for Integrated Cell-Material Sciences (WPI-iCeMS), Institute for Advanced Study, Kyoto University, Yoshida, Sakyo-ku, Kyoto, 606-8501 Japan

Correction to: *Scientific Reports* 10.1038/s41598-017-18108-0, published online 18 December 2017

The original version of this Article contained a typographical error in the spelling of the author Sanjog S. Nagarkar, which was incorrectly given as Sanjog Nagarkar. This has now been corrected in the PDF and HTML versions of the Article, and in the accompanying Supporting Information file.

