# Supplementary material for: Chemical Adsorption and Physical Confinement of Polysulfides with the Janus-faced Interlayer for High-performance Lithium-Sulfur Batteries
Source: Sci Rep. 2017 Dec 18;7:17703. doi: 10.1038/s41598-017-18108-0 (PMC5735181; doi:10.1038/s41598-017-18108-0)
Supplement: Supplementary file 1 — Supporting information [file 41598_2017_18108_MOESM1_ESM.pdf]

# Supporting Information

## Chemical Adsorption and Physical Confinement of Polysulfides with the Janus-faced Interlayer for High-performance Lithium-Sulfur Batteries

Poramane Chiochan,[1] Siriroong Kaewruang,[1] Nutthaphon Phattharasupakun,[1] Juthaporn Wutthiprom,[1] Thana Maihom,[1] Jumras Limtrakul,[2] Sanjog S. Nagarkar,[3] Satoshi Horike,[3] and Montree Sawangphruk[1,*]

[1]Department of Chemical and Biomolecular Engineering, School of Energy Science and Engineering, Vidyasirimedhi Institute of Science and Technology, Rayong 21210, Thailand.
[2]Department of Materials Engineering, School of Molecular Science and Engineering, Vidyasirimedhi Institute of Science and Technology, Rayong 21210, Thailand.
[3]Institute for Integrated Cell-Material Sciences (WPI-iCeMS), Institute for Advanced Study, Kyoto University, Yoshida, Sakyo-ku, Kyoto 606-8501, Japan.

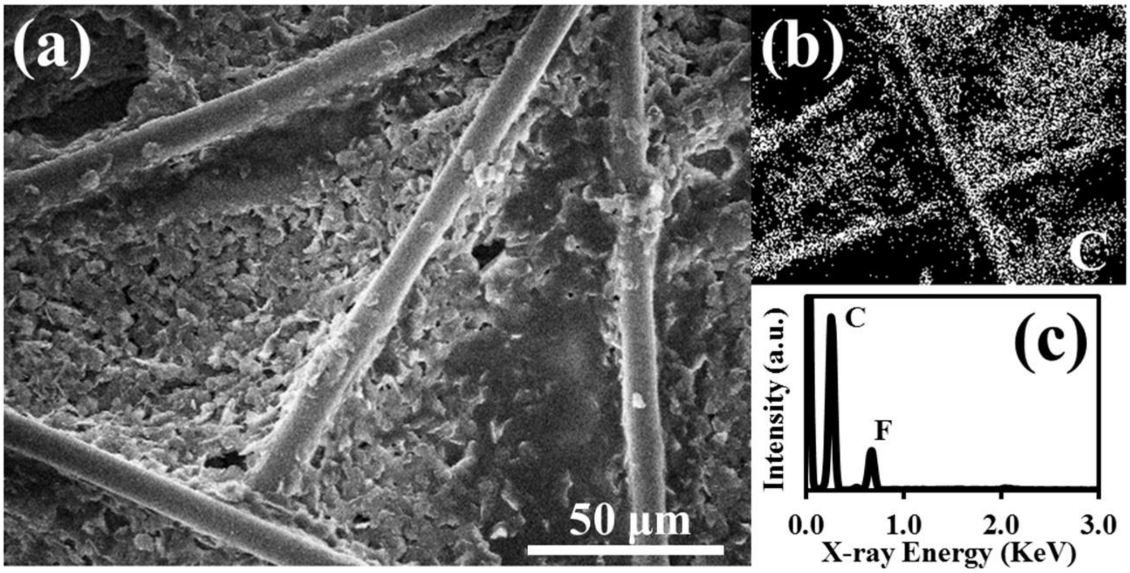


**Figure S1.** (a) FESEM image, (b) EDX mapping, and (c) EDX spectrum of CFP.

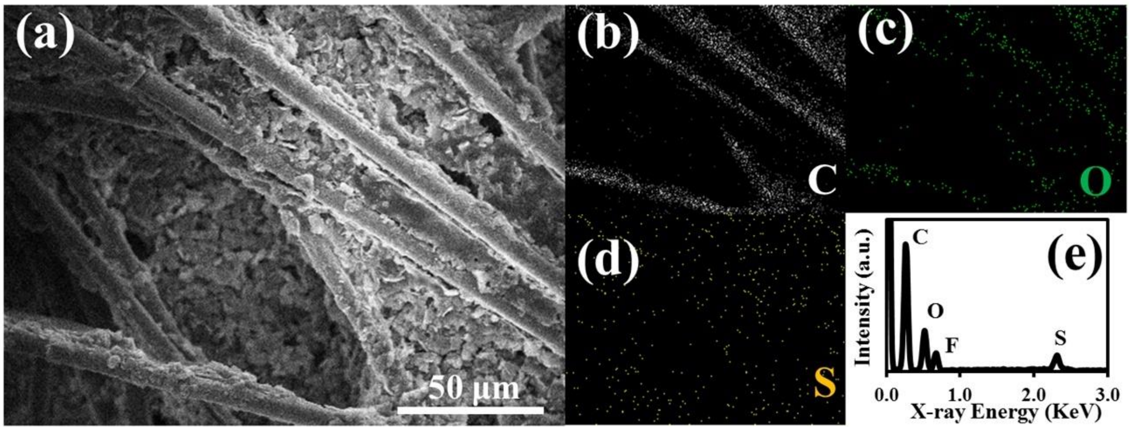


**Figure S2**. (a) FESEM image, (b-d) EDX mapping, and (e) EDX spectrum of *f*-CFP.

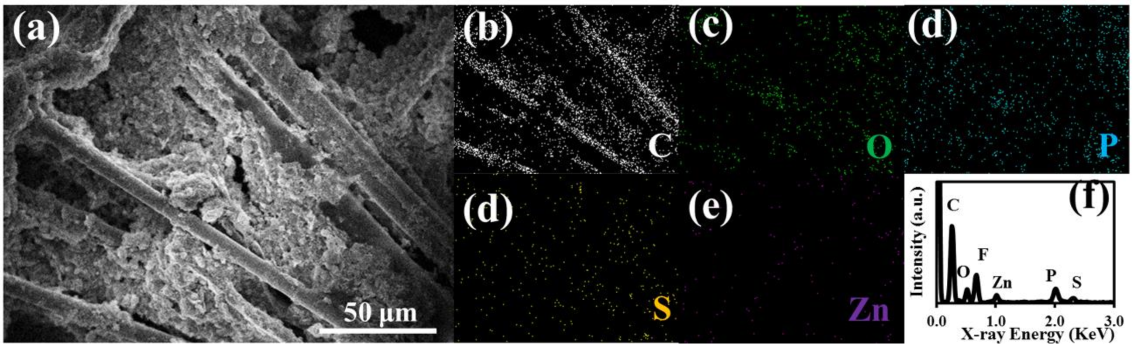


**Figure S3**. (a) FESEM image, (b-e) EDX mapping, and (f) EDX spectrum of ZnPTz coated on *f*-CFP.

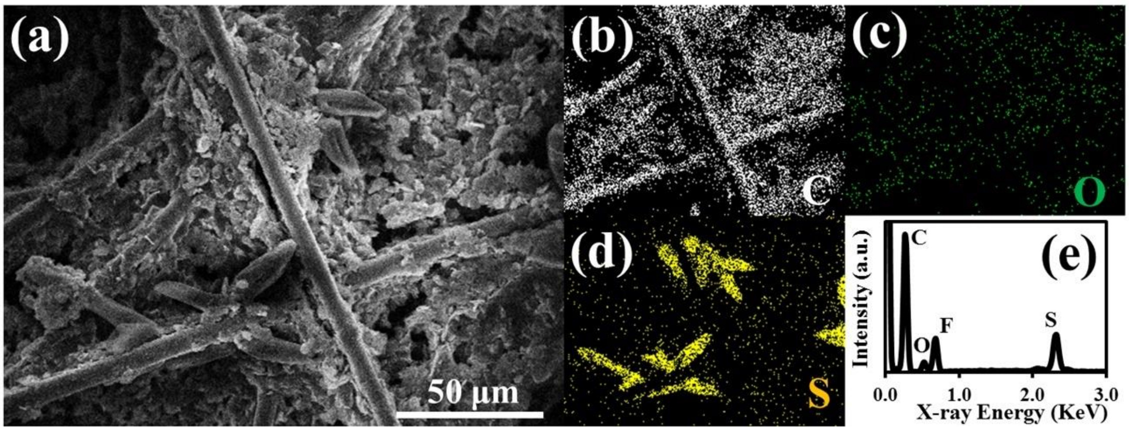


**Figure S4.** (a) FESEM image, (b-d) EDX mapping, and (e) EDX spectrum of as-cycled *f*-CFP placed near the separator side.

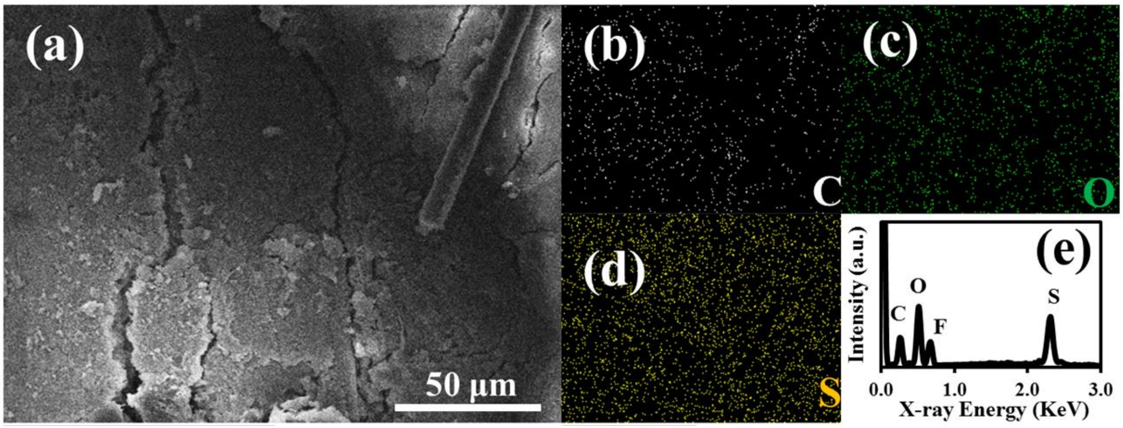


**Figure S5.** (a) FESEM image, (b-d) EDX mapping, and (e) EDX spectrum of cycled *f*-CFP placed near the cathode side.

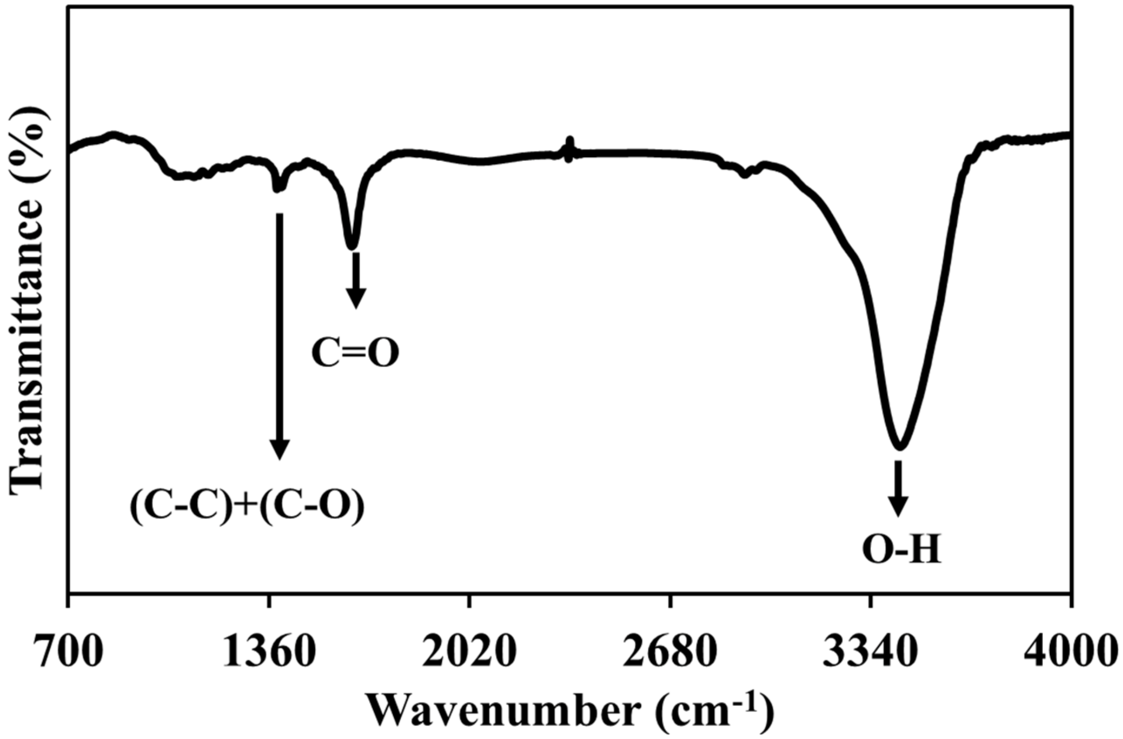


**Figure S6.** FTIR spectrum of *f*-CFP.

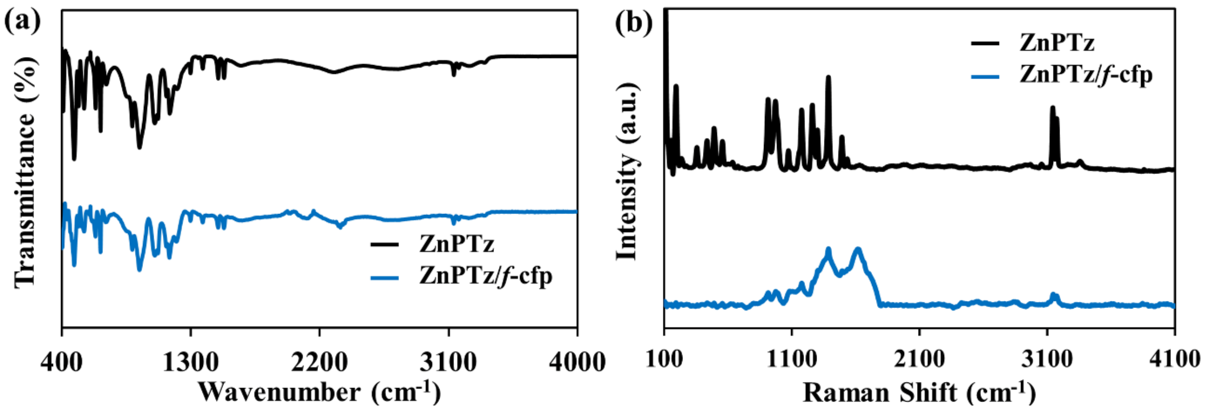


**Figure S7.** (a) FTIR and (b) Raman spectra of ZnPTz powder (black line) and ZnPTz coated on *f*-CFP interlayer (blue line).

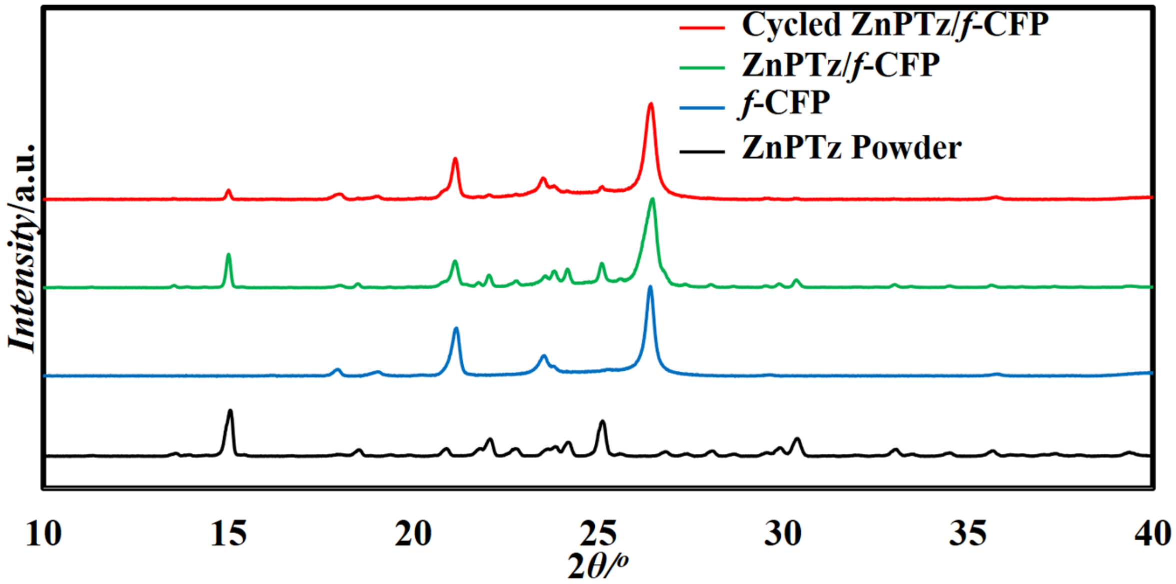


**Figure S8.** The XRD pattern of ZnPTz powder, *f*-CFP, ZnPTz/*f*-CFP, Cycled ZnPTz/*f*-CFP

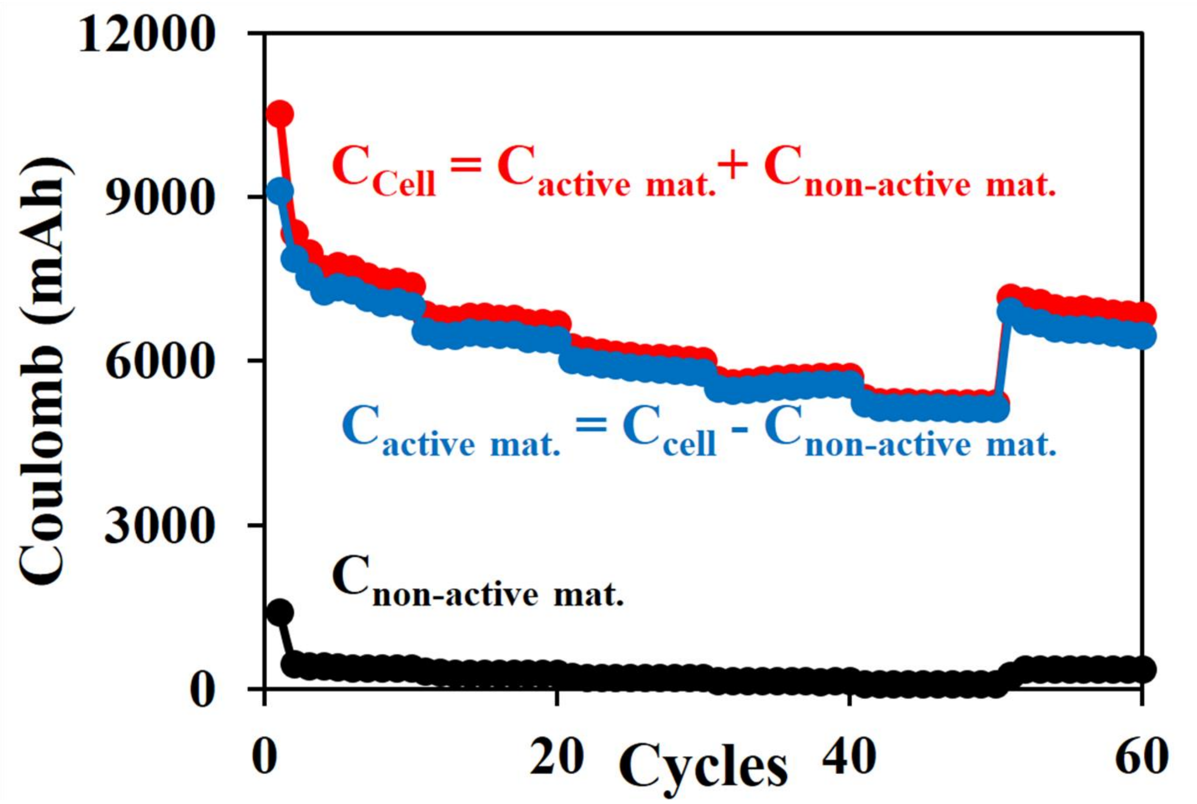


**Figure S9.** The rate capability of ZnPTz/*f*-CFP interlayer without sulfur cathode.

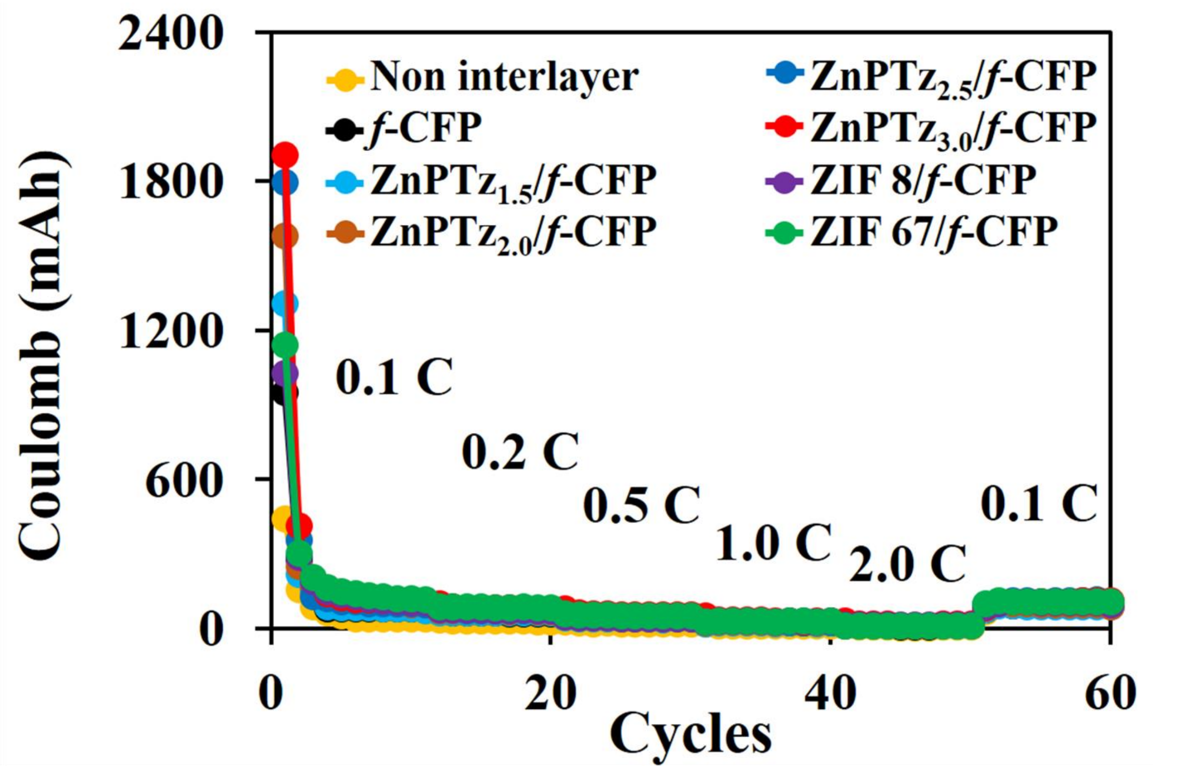


**Figure S10.** The rate performance of non-related active materials.

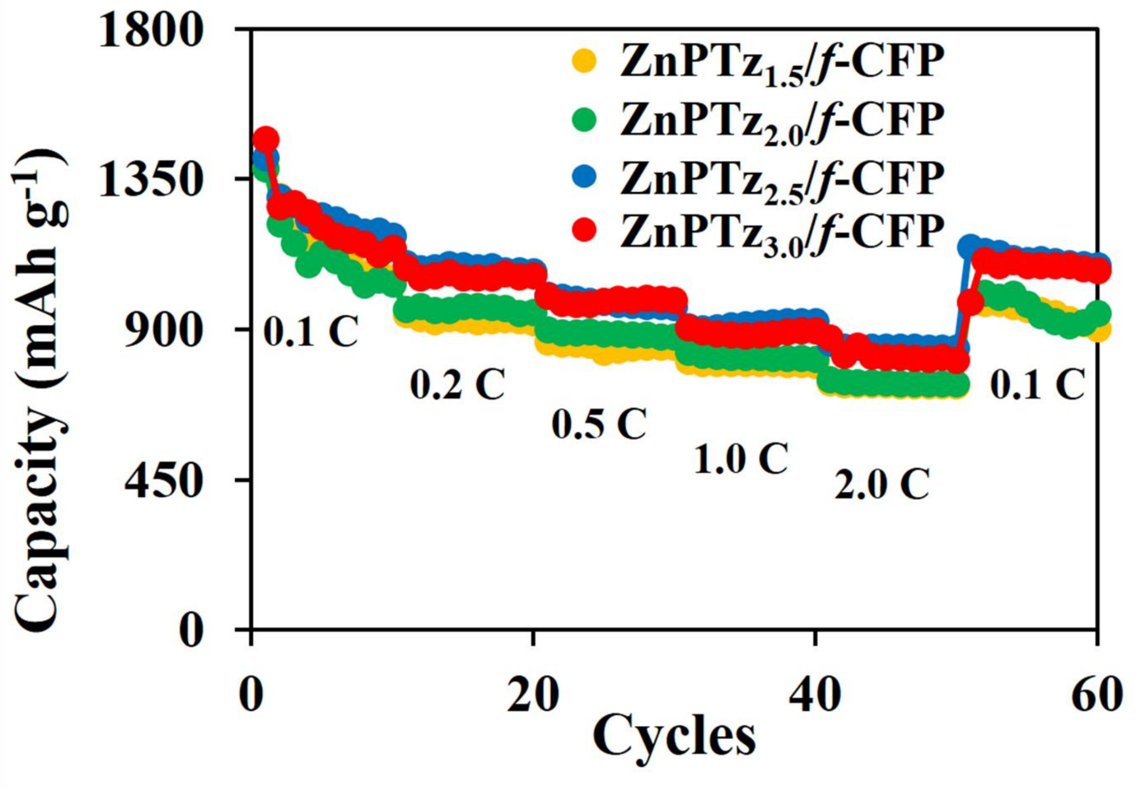


**Figure S11.** The rate performance of LSBs with different areal masses of ZnPTz on *f*-CFP interlayer.

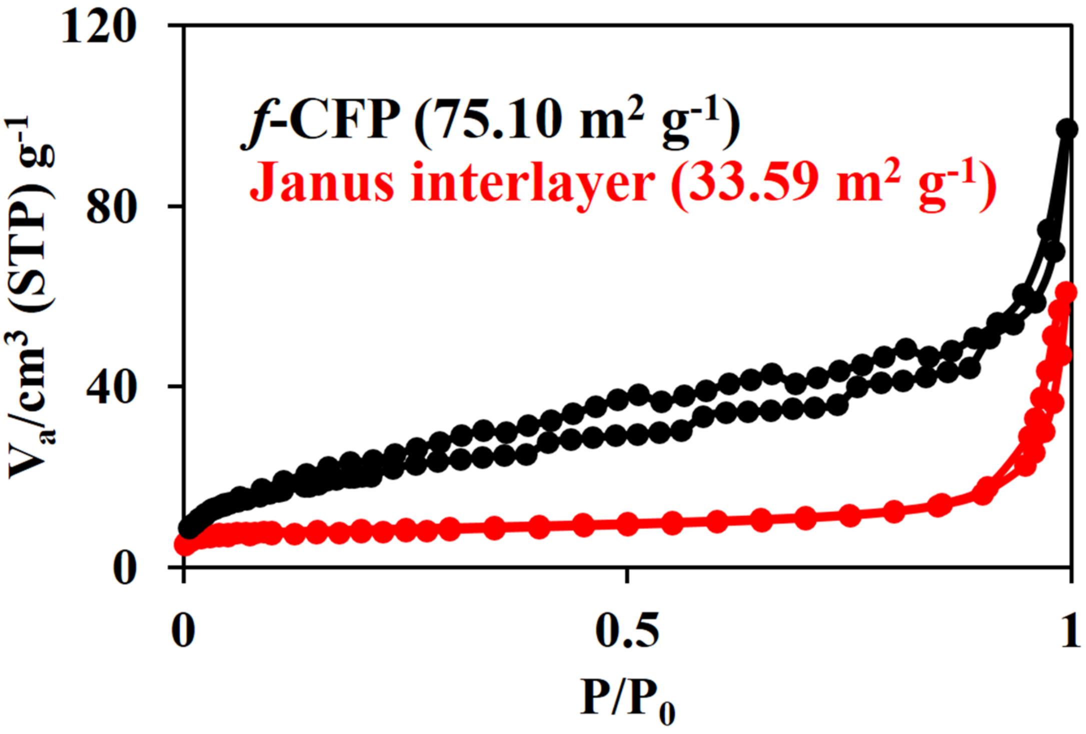


**Figure S12**. $N_2$ adsorption-desorption isotherm of *f*-CFP interlayer and ZnPTz/*f*-CFP Janus interlayer.

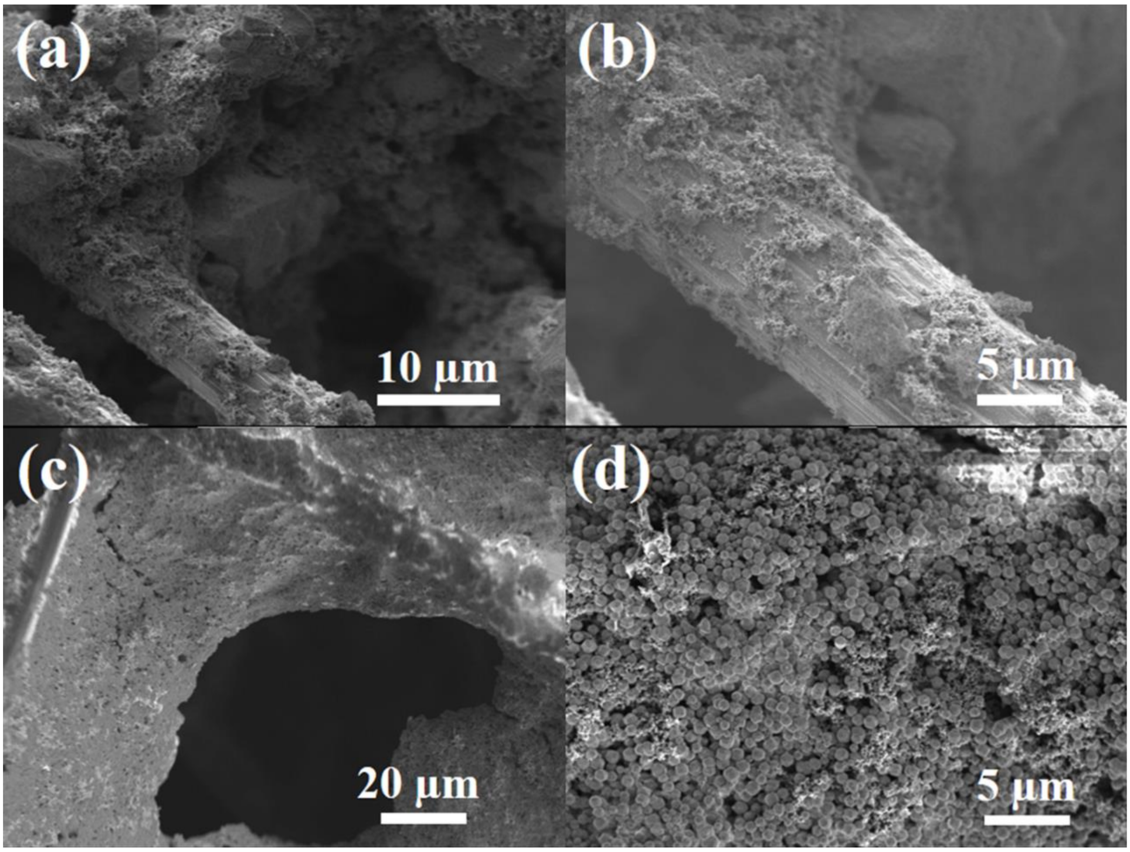


**Figure S13.** FESEM images of (a and b) ZIF-8/*f*-CFP interlayer and (c and d) ZIF-67/*f*-CFP interlayer.

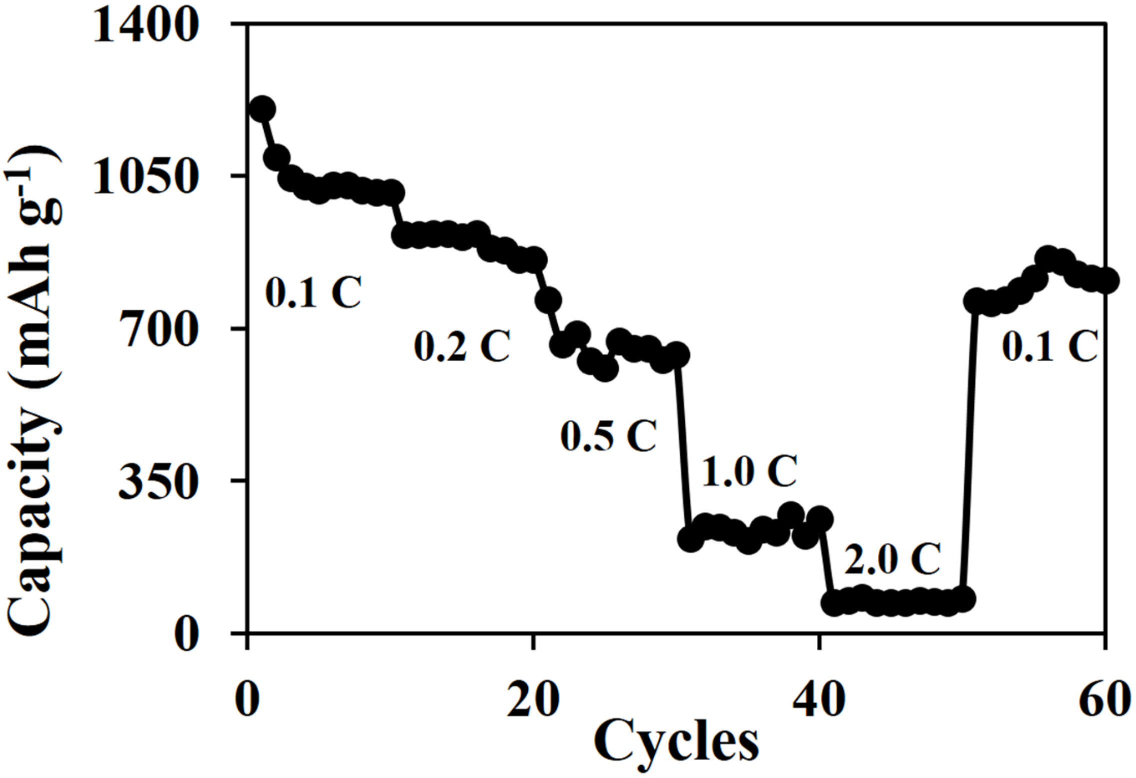


**Figure S14.** The rate performance of LSB using a high-loading active material (9.57 $mg_{Sulfur}$ $cm^{-2}$ cathode or 19.23 S a cell) with the ZnPTz/*f*-CFP interlayer.

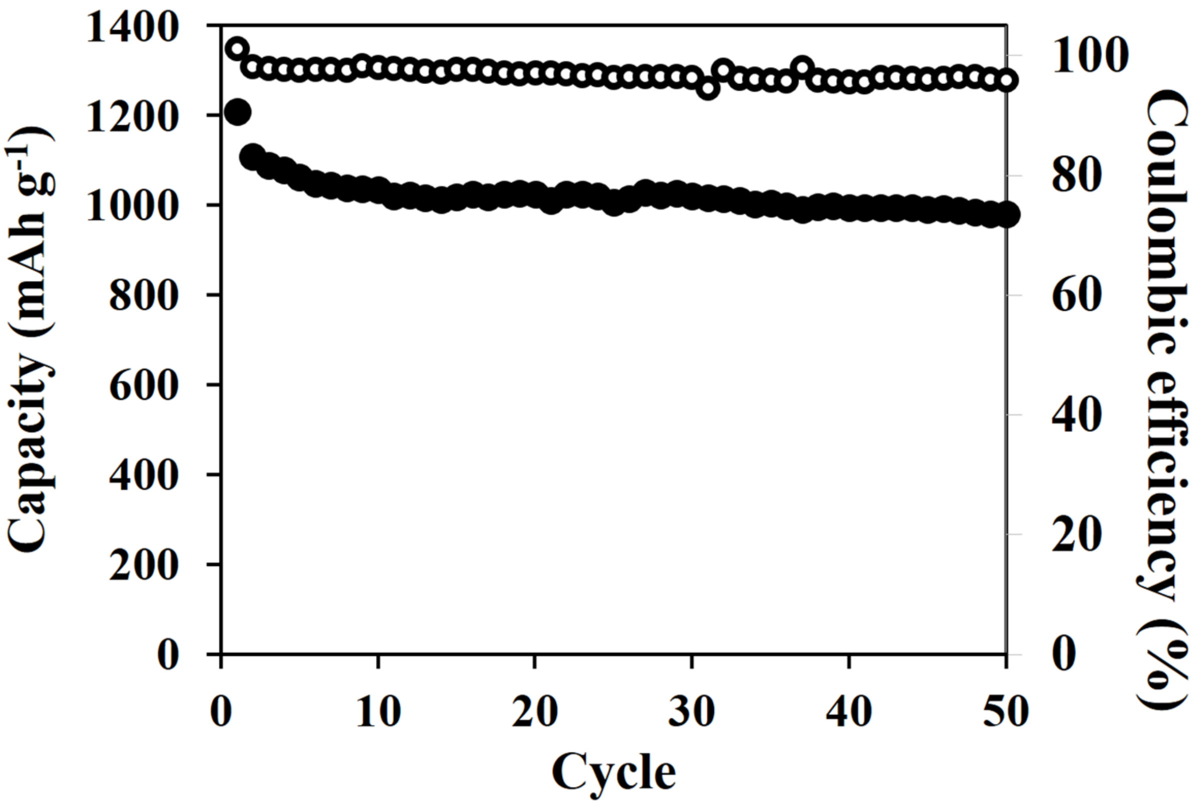


**Figure S15.** Cycling performance of LSB using 9.57 $mg_{Sulfur}$ $cm^{-2}$ cathode (19.23 mg S a cell) with the ZnPTz/*f*-CFP interlayer at 0.1 C for 50 cycles.

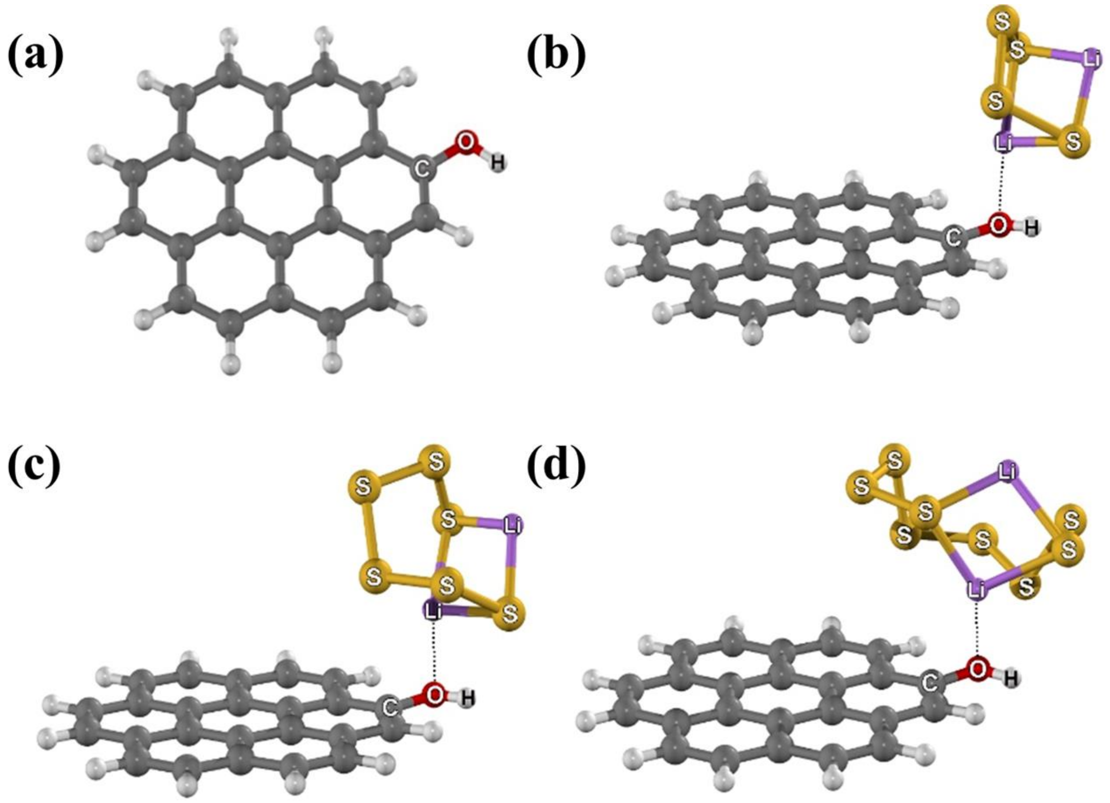


**Figure S16.** Optimized structures of the CFP-OH model (a) and its interaction with (b) $Li_2S_4$, (c) $Li_2S_6$, and (d) $Li_2S_8$.

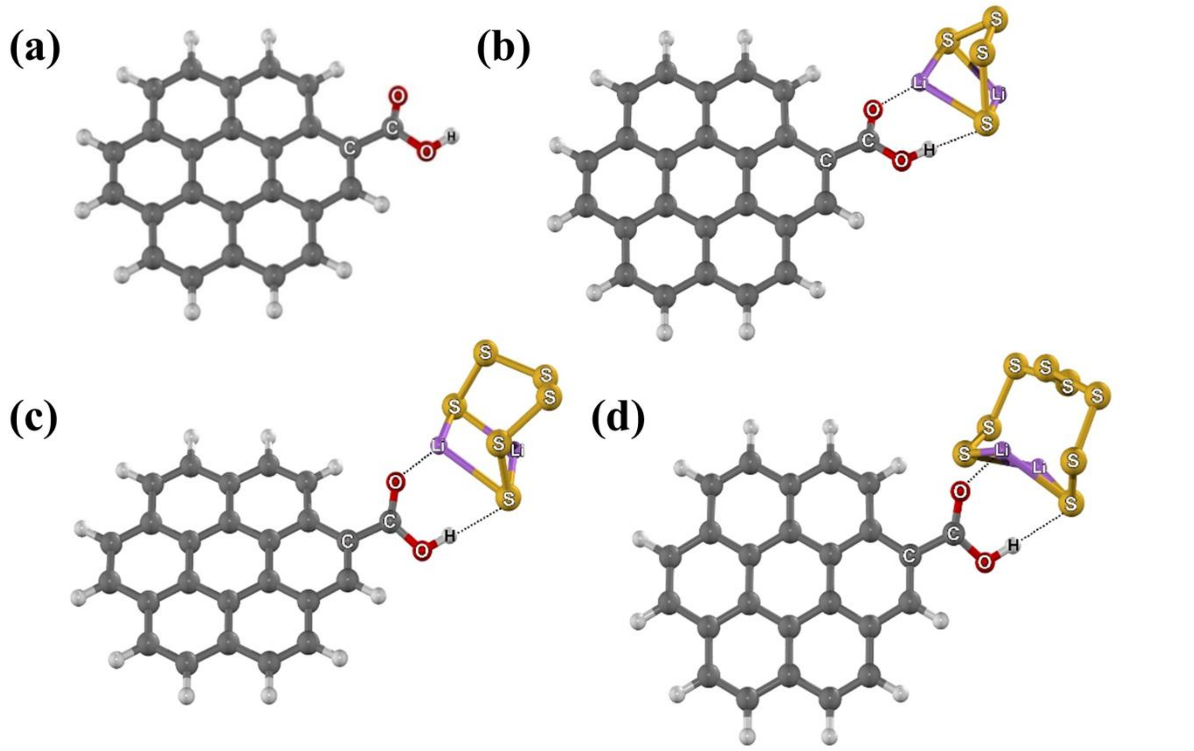


**Figure S17.** Optimized structures of the CFP-COOH model (a) and its interaction with (b) $Li_2S_4$, (c) $Li_2S_6$, and (d) $Li_2S_8$.

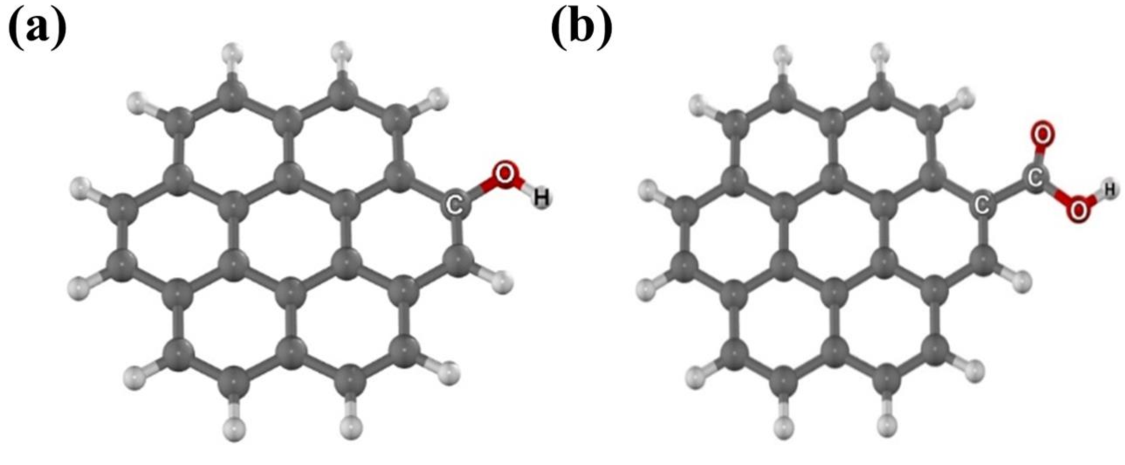


**Figure S18.** The structural models of (a) *f*-CFP-OH and (b) *f*-CFP-COOH.

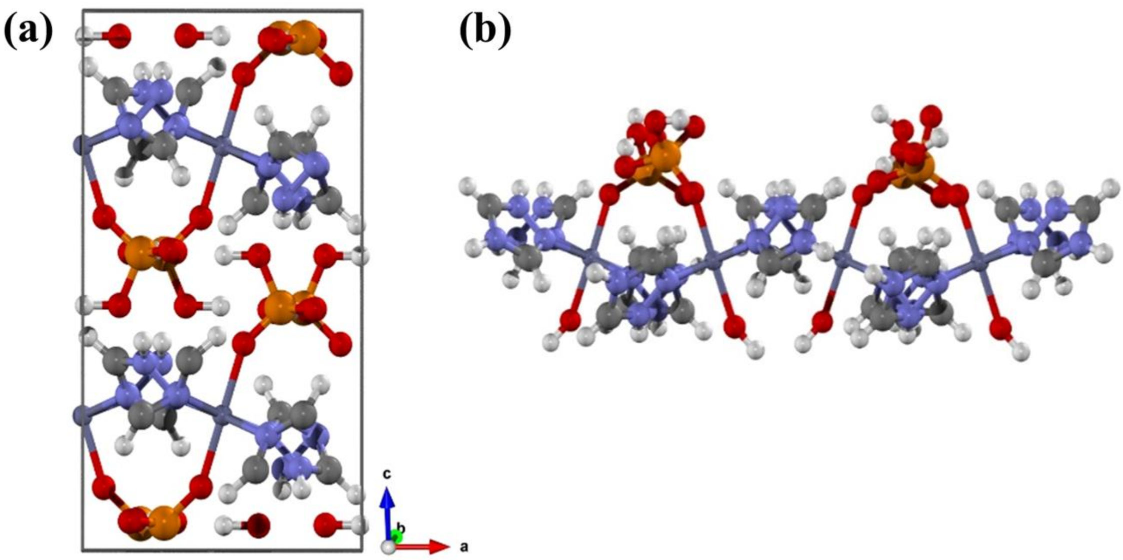


**Figure S19.** (a) Unit cell and (b) 001 surface cluster model of $[Zn\text{-}(H_2PO_4)_2(TzH)_2]_n$ 2D coordination network used in this work. Zn, P, O, N, C, H are light grey, orange, red, blue, gray, and white, respectively.

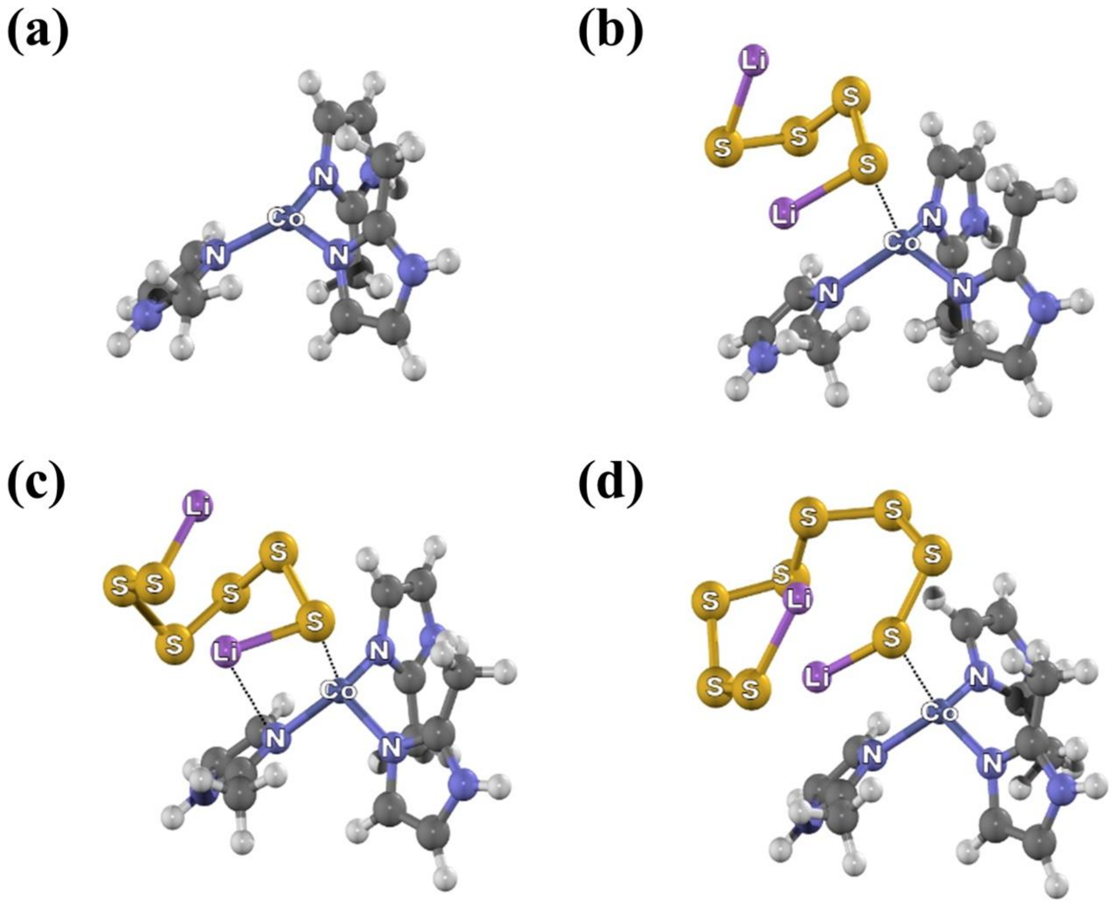


**Figure S20.** Optimized structures of ZIF-67 cluster (a) and its interaction with (b) $Li_2S_4$, (c) $Li_2S_6$, and (d) $Li_2S_8$.

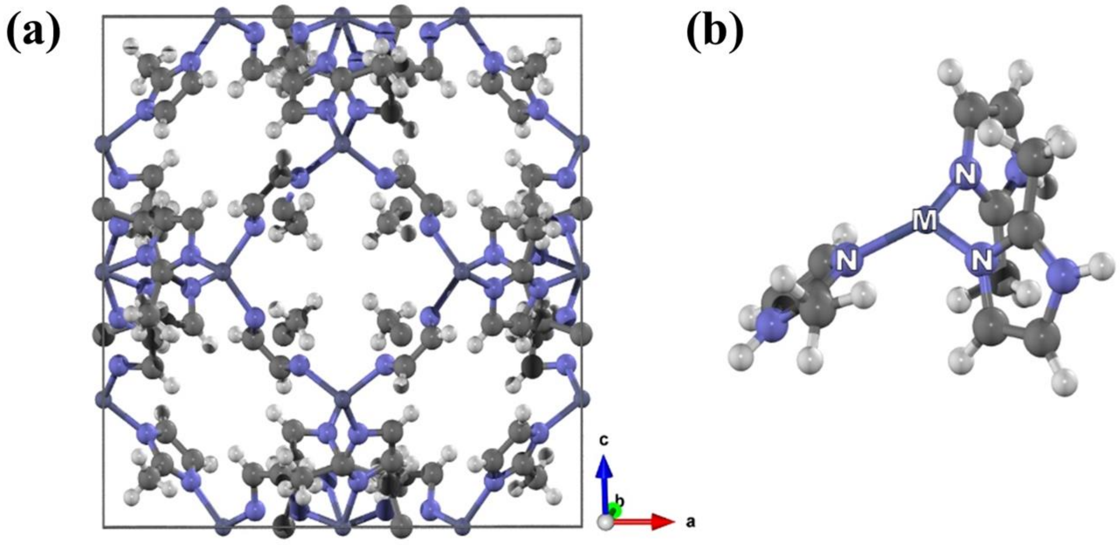


**Figure S21.** (a) Unit cell and (b) surface cluster model of ZIF-8 or ZIF-67 used in this work. M refers to Zn for ZIF-8 or Co for ZIF-67.

**Table S1** Binding energies of the LiPS species on the coordination network and *t*-CFP materials.

| Lithium Polysulfides (LiPSs) | Binding energy (eV) | | | | |
|---|---|---|---|---|---|
| | **[Zn-($H_2PO_4$)$_2$(TzH)$_2$]$_n$** | **ZIF-8** | **ZIF-67** | ***t*-CFP-OH** | ***t*-CFP-COOH** |
| **$Li_2S_4$** | **-2.56** | **-1.47** | **-1.57** | **-1.07** | **-0.71** |
| **$Li_2S_6$** | **-2.38** | **-1.44** | **-1.63** | **-1.03** | **-0.78** |
| **$Li_2S_8$** | **-2.22** | **-1.39** | **-1.52** | **-1.10** | **-0.91** |

## References

1. D. Umeyama, S. Horike, M. Inukai, T. Itakura and S. Kitagawa, *J. Am. Chem. Soc.*, 2012, **134**, 12780-12785.
2. P. Chiochan, N. Phattharasupakun, J. Wutthiprom, M. Suksomboon, S. Kaewruang, P. Suktha and M. Sawangphruk, *Electrochim. Acta*, 2017, **237**, 78-86.
3. P. Suktha, P. Chiochan, P. Iamprasertkun, J. Wutthiprom, N. Phattharasupakun, M. Suksomboon, T. Kaewsongpol, P. Sirisinudomkit, T. Pettong and M. Sawangphruk, *Electrochim. Acta*, 2015, **176**, 504-513.
4. M. Sawangphruk, M. Suksomboon, K. Kongsupornsak, J. Khuntilo, P. Srimuk, Y. Sanguansak, P. Klunbud, P. Suktha and P. Chiochan, *J. Mater. Chem. A*, 2013, **1**, 9630-9636.
5. Y. Zhao and D. G. Truhlar, *Theoretical Chemistry Accounts*, 2008, **120**, 215-241.
6. Y. H. Shao, Z. T. Gan, E. Epifanovsky, A. T. B. Gilbert, M. Wormit, J. Kussmann, A. W. Lange, A. Behn, J. Deng, X. T. Feng, D. Ghosh, M. Goldey, P. R. Horn, L. D. Jacobson, I. Kaliman, R. Z. Khaliullin, T. Kus, A. Landau, J. Liu, E. I. Proynov, Y. M. Rhee, R. M. Richard, M. A. Rohrdanz, R. P. Steele, E. J. Sundstrom, H. L. Woodcock, P. M. Zimmerman, D. Zuev,

B. Albrecht, E. Alguire, B. Austin, G. J. O. Beran, Y. A. Bernard, E. Berquist, K. Brandhorst, K. B. Bravaya, S. T. Brown, D. Casanova, C. M. Chang, Y. Q. Chen, S. H. Chien, K. D. Closser, D. L. Crittenden, M. Diedenhofen, R. A. DiStasio, H. Do, A. D. Dutoi, R. G. Edgar, S. Fatehi, L. Fusti-Molnar, A. Ghysels, A. Golubeva-Zadorozhnaya, J. Gomes, M. W. D. Hanson-Heine, P. H. P. Harbach, A. W. Hauser, E. G. Hohenstein, Z. C. Holden, T. C. Jagau, H. J. Ji, B. Kaduk, K. Khistyaev, J. Kim, J. Kim, R. A. King, P. Klunzinger, D. Kosenkov, T. Kowalczyk, C. M. Krauter, K. U. Lao, A. D. Laurent, K. V. Lawler, S. V. Levchenko, C. Y. Lin, F. Liu, E. Livshits, R. C. Lochan, A. Luenser, P. Manohar, S. F. Manzer, S. P. Mao, N. Mardirossian, A. V. Marenich, S. A. Maurer, N. J. Mayhall, E. Neuscamman, C. M. Oana, R. Olivares-Amaya, D. P. O'Neill, J. A. Parkhill, T. M. Perrine, R. Peverati, A. Prociuk, D. R. Rehn, E. Rosta, N. J. Russ, S. M. Sharada, S. Sharma, D. W. Small, A. Sodt, T. Stein, D. Stuck, Y. C. Su, A. J. W. Thom, T. Tsuchimochi, V. Vanovschi, L. Vogt, O. Vydrov, T. Wang, M. A. Watson, J. Wenzel, A. White, C. F. Williams, J. Yang, S. Yeganeh, S. R. Yost, Z. Q. You, I. Y. Zhang, X. Zhang, Y. Zhao, B. R. Brooks, G. K. L. Chan, D. M. Chipman, C. J. Cramer, W. A. Goddard, M. S. Gordon, W. J. Hehre, A. Klamt, H. F. Schaefer, M. W. Schmidt, C. D. Sherrill, D. G. Truhlar, A. Warshel, X. Xu, A. Aspuru-Guzik, R. Baer, A. T. Bell, N. A. Besley, J. D. Chai, A. Dreuw, B. D. Dunietz, T. R. Furlani, S. R. Gwaltney, C. P. Hsu, Y. S. Jung, J. Kong, D. S. Lambrecht, W. Z. Liang, C. Ochsenfeld, V. A. Rassolov, L. V. Slipchenko, J. E. Subotnik, T. Van Voorhis, J. M. Herbert, A. I. Krylov, P. M. W. Gill and M. Head-Gordon, *Mol. Phys.*, 2015, **113**, 184-215.

7. P. J. Hay and W. R. Wadt, *J. Chem. Phys.*, 1985, **82**, 270-283.
8. C. Zhang, C. Han, D. S. Sholl and J. R. Schmidt, *J. Phys. Chem. Lett.*, 2016, **7**, 459-464.
9. Z. Wang, Y. Dong, H. Li, Z. Zhao, H. Bin Wu, C. Hao, S. Liu, J. Qiu and X. W. Lou, *Nat. Commun.*, 2014, **5**, 5002.
10. A. E. Reed, L. A. Curtiss and F. Weinhold, *Chem. Rev.*, 1988, **88**, 899-926.
